# Arginine metabolism supports *de novo* pyrimidine biosynthesis to block DNA damage and maintain Epstein-Barr virus latency

**DOI:** 10.1128/mbio.00933-26

**Published:** 2026-06-15

**Authors:** Shaowen White, Yifei Liao, Yin Wang, Shunji Li, Eric M. Burton, John M. Asara, Benjamin E. Gewurz

**Affiliations:** 1 Division of Infectious Diseases, Department of Medicine, Brigham and Women's Hospital 1861, Boston, Massachusetts, USA; 2 Broad Institute 33577, Cambridge, Massachusetts, USA; 3 Division of Signal Transduction, Department of Medicine, Beth Israel Deaconess Medical Center 1859 , Boston, Massachusetts, USA; Tsinghua University, Beijing, China

**Keywords:** viral latency, lytic cycle, reactivation, metabolism, epigenetic, DNA damage, *de novo* pyrimidine synthesis, nucleotide biosynthesis, nucleotide metabolism, double-stranded DNA virus

## Abstract

Incompletely understood mechanisms serve to maintain Epstein-Barr virus (EBV) latency, in which viral oncogene(s) are expressed, but lytic antigens are not expressed. Shortly after the discovery of EBV and even before it was named, Werne and Gertrude Henle identified that restriction of extracellular arginine induces EBV lytic antigens within Burkitt lymphoma tumor cells. However, for nearly 60 years, it has remained unknown how arginine metabolism supports EBV latency. To gain insights, we performed an amino acid restriction screen in EBV+ Burkitt cell lines. This confirmed that arginine restriction was sufficient to trigger EBV reactivation in Burkitt B cells and in gastric carcinoma models. Arginine restriction strongly impaired *de novo* pyrimidine biosynthesis, and CRISPR- or chemical genetic-blockade of pyrimidine biosynthesis enzymes induced EBV immediate-early and early lytic gene expression. However, arginine restriction blocked EBV lytic DNA replication and, consequently, also late gene expression, suggesting an abortive lytic cycle. By contrast, chemical or CRISPR blockade of the *de novo* pyrimidine biosynthesis pathway rate-limiting enzyme CAD reactivated a full EBV lytic cycle, suggesting specific arginine restriction roles in support of lytic DNA replication. Arginine restriction caused DNA damage, which was a driver of EBV reactivation. Arginine restriction and DNA hypomethylation additively reactivated EBV. Together, our results highlight arginine and pyrimidine metabolism as potential targets for EBV lytic antigen induction therapy in B-cell and epithelial-cell contexts.

**IMPORTANCE:**

Altered metabolism is a hallmark of cancer, frequently increasing transformed cell dependence on extracellular amino acid supply. Despite current interest in Epstein-Barr virus (EBV) lytic antigen induction therapy, in which viral lytic reactivation sensitizes tumors to the highly cytotoxic effects of the antiviral ganciclovir, there has been no systematic study of extracellular amino acids that control EBV latency. We identified that arginine uptake was important for the maintenance of EBV latency in both Burkitt lymphoma and gastric carcinoma contexts. Metabolic pathway analyses highlighted that arginine uptake and metabolism were required to supply pyrimidines. Disruption of arginine metabolism or de novo pyrimidine synthesis caused DNA damage. Arginine restriction also triggered Burkitt DNA hypermethylation. Building upon this, we provide evidence that the combination of arginine restriction and DNA hypomethylation, either by decitabine or by CRISPR approaches, induced EBV reactivation more strongly than either alone, suggesting a therapeutic approach..

## INTRODUCTION

The gamma-herpesvirus Epstein-Barr virus (EBV) chronically infects over 95% of adults worldwide (1). EBV was the first human tumor virus discovered through its close association with the endemic Burkitt lymphoma, which remains the most common pediatric non-Hodgkin B-cell lymphoma in areas with holoendemic malaria (2, 3). EBV contributes to ~1.5% of all human cancers, including multiple subtypes of both Hodgkin and non-Hodgkin B-cell lymphomas, natural killer and T-cell lymphomas, and gastric and nasopharyngeal carcinomas (2, 4–7). In most infected tumor cells, EBV maintains a state of viral latency, in which nearly 80 viral lytic cycle genes are repressed.

EBV infects both lymphocytes and epithelial cells to persistently infect hosts and spread between individuals. EBV uses a series of latency genes to traverse the B-cell compartment and colonize the memory B-cell reservoir (8). EBV latency programs, which express between one and nine viral oncoproteins and EBV non-coding RNAs, expand the infected B-cell pool and enable infected cells to differentiate into long-lived memory B cells (8–10). Within latently infected cells, the double-stranded DNA viral genome is maintained as circular, extrachromosomal episomes.

Memory B-cell differentiation into plasma cells is thought to be the major physiological B-cell trigger for EBV reactivation. Within Burkitt tumor cells, which are a major model for studies of EBV reactivation as robust primary cell models of EBV reactivation have yet to be developed, B-cell immunoglobulin receptor cross-linking triggers reactivation, as does treatment with the tumor-promoting agent (also called phorbol myristate acetate) and the histone deacetylase inhibitor sodium butyrate (11, 12). Many EBV-infected tumor cells, including Burkitt and gastric carcinoma cells, exhibit high levels of DNA CpG hypermethylation, which support viral latency. Upon lytic reactivation, the EBV-encoded immediate-early genes BZLF1 and BRLF1 are the first to be expressed, which are transcription activators that induce expression of ~35 early lytic viral genes (13, 14). These include factors required for lytic-cycle EBV genome amplification, including the processivity factor BMRF1 and the protein kinase BGLF4 (15–20). Newly synthesized, linear, unchromatinized viral genomes serve as templates for expression of ~37 EBV late genes (21–23), which include virion structural proteins, including the capsid protein p18 and virion glycoproteins, including glycoprotein 350 (gp350) (24, 25). BGLF4 sensitizes lytic cells and their neighbors to the highly cytotoxic activity of the antiviral nucleoside ganciclovir (26, 27). EBV lytic antigens also promote potent innate and adaptive immune responses (15, 28, 29). Consequently, there is significant interest in the development of strategies to induce EBV lytic reactivation to sensitize EBV+ tumors to ganciclovir and immunotherapy approaches (30–32).

Altered metabolism is a hallmark of cancer (33). In fact, Burkitt B cells are among the fastest growing human tumors (34). Cancer cells frequently rewire major central carbon metabolism pathways to support aerobic glycolysis (the so-called Warburg effect) (35), increased dependency on *de novo* nucleotide biosynthesis (36), extracellular arginine (37), asparagine (38), cysteine (39), and methionine (40). Shortly after EBV was discovered but before it was named, Werner and Gertrude Henle serendipitously discovered that restriction of extracellular arginine reactivated expression of several EBV lytic antigens against which antisera were available in newly established human tumor-derived Burkitt lymphoma cell lines (41). However, the mechanism was not pursued at that early period of Burkitt tumor research, and key questions have remained unaddressed, such as the extent to which arginine restriction induces EBV lytic antigen expression, how it does so, and in which latently infected host cellular contexts this phenomenon occurs.

Arginine is a conditionally essential amino acid whose *de novo* synthesis in the small intestine and kidney meets demand in healthy adults, but it is essential (must be acquired through the diet) in infants, growing children, and adults under catabolic stress or with dysfunction of the small intestine or kidney (42, 43). Its metabolism is a vulnerability of many types of cancer cells, especially those in which the gene encoding argininosuccinate synthase (ASS1), the rate-limiting enzyme in the arginine biosynthesis pathway (44, 45), has been epigenetically silenced. In addition to its obligatory role in protein biosynthesis, arginine metabolism yields a range of important metabolites (46) that serve as major regulators of mitochondrial physiology, including respiration and mitochondrial biogenesis (47–49), and nucleotide metabolism. Arginine supports eukaryotic pyrimidine *de novo* biosynthesis, which in turn drives the growth and proliferation of EBV-transformed B cells (50). Notably, halogenated pyrimidine analogs such as 5-fluorouracil and idoxuridine, and the antifolate methotrexate, each trigger EBV lytic gene expression (51–54). Methotrexate disrupts tetrahydrofolate synthesis, which is required for *de novo* purine biosynthesis (55). We also recently reported that purine biosynthesis inhibition reactivates EBV lytic antigen expression (56). These observations suggest that nucleotide metabolism supports EBV latency through incompletely defined mechanisms.

The *de novo* pyrimidine biosynthesis pathway relies on a series of enzymes, including the rate-limiting enzyme CAD (carbamoyl-phosphate synthetase II, aspartate transcarbamoylase, and dihydroorotase), UMPS (uridine 5′-monophosphate synthase), and the mitochondrial membrane protein DHODH (dihydroorotate dehydrogenase). CAD mediates the biosynthesis of dihydroorotate from carbamoyl phosphate and aspartate. DHODH and UMPS then mediate downstream biosynthesis of uridine 5′-monophosphate (UMP). These proteins form a complex called the pyrimidinosome that spans the inner and outer mitochondrial membranes (57). Pyrimidinosome activity is regulated by AMP-activated protein kinase, whose activity is in turn regulated by the AMP/ATP and ADP/ATP ratios (57, 58) or by mitochondrial electron transport chain activity (59). In bacteria, *de novo* pyrimidine synthesis activity is regulated by arginine levels, as both arginine and pyrimidine biogenesis consume carbamoyl phosphate (60), suggesting that arginine and pyrimidine biosynthesis are evolutionarily related pathways.

To systematically identify amino acids that contribute to EBV latency, we conducted an amino acid restriction screen, which confirmed arginine’s essential role in EBV latency maintenance. Metabolomic analyses revealed that extracellular arginine is required for Burkitt pyrimidine *de novo* biosynthesis. Restriction of extracellular arginine disrupted *de novo* pyrimidine biosynthesis at the level of aspartate transcarbamoylation, and perturbation of *de novo* pyrimidine biosynthesis therefore also triggered EBV lytic reactivation. Arginine restriction-mediated EBV reactivation required host cell DNA damage response pathways and additively drove EBV lytic reactivation together with DNA hypomethylation.

## RESULTS

### An amino acid restriction screen for Burkitt lymphoma EBV lytic reactivation

**Fig 1:**
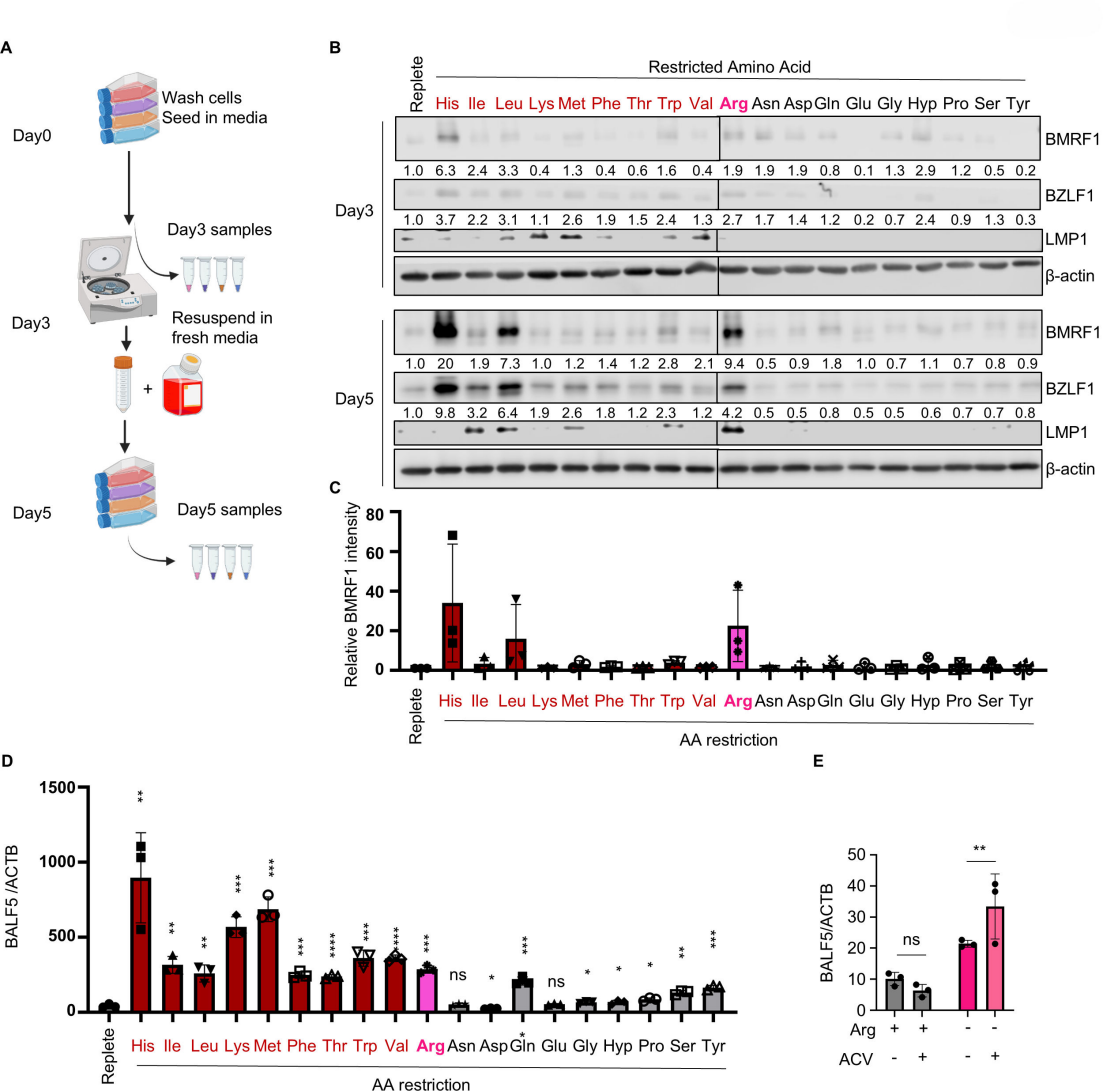
Screen of amino acid restriction effects on EBV+ Burkitt B-cell EBV reactivation. (**A**) Schematic of amino acid restriction of P3HR-1 Burkitt cells. (**B**) Immunoblot analysis of whole-cell lysates (WCL) from P3HR-1 grown in replete media vs in media restricted for the indicated amino acid for 3 or 5 days. Blots are representative of *n* = 3 replicates. (**C**) Densitometry quantification of β-actin normalized BMRF1 band intensity. Shown are mean ± SD values from *n* = 3 replicates as in panel **B**, normalized to BMRF1 levels in cells grown in replete media, which were set to a value of 1. (**D**) Analysis of amino acid restriction effects on P3HR-1 EBV genome copy number. Shown are the mean ± SD EBV intracellular genome copy number from qPCR analysis of *n* = 3 biological replicates of P3HR-1 grown in replete or arginine-free media for 5 days. Values were calculated by PCR analysis of EBV genome BALF5 normalized to host genome β-actin (ACTB) signals. (**E**) Mean ± SD EBV intracellular genome copy number as in panel **D** from cells grown in replete or arginine-free conditions for 5 days with or without 100 μg/mL acyclovir (ACV) to block EBV lytic replication. Student’s *t*-test was performed for the indicated values compared to those from cells grown in replete media. ****P < 0.0001, ***P < 0.001, **P < 0.01, and *P < 0.05.

To systematically test whether the supply of particular amino acids is necessary for EBV latency, we performed an amino acid restriction screen in EBV+ P3HR-1 Burkitt lymphoma cells. To do so, we grew cells for 5 days in RPMI media lacking one of each of the 20 amino acids, together with fetal calf serum (FCS) dialyzed to remove free amino acids (Fig. 1A). Restriction of any of the nine essential amino acids led to cell growth arrest (Fig. S1A). Restriction of non-essential amino acids had a comparatively little effect on Burkitt proliferation, as expected, with the exception of arginine, cystine (the oxidized form of cysteine present in media), glutamine, and tyrosine (Fig. S1B). Cystine restriction resulted in widespread cell death by day 3, likely due to its obligatory role in redox defense against lipid peroxidation (61). Thus, cystine restriction was omitted from further analyses.

To define amino acid restriction effects on EBV latency, immunoblot analysis was performed on whole-cell lysates (WCL) of EBV+ P3HR-1 following 3 or 5 days of incubation in media restricted for one of each amino acid. Restriction of methionine or lysine induced the EBV oncoprotein LMP1, which is expressed by the EBV latency IIa and III programs and also by the lytic cycle. To a lesser degree, these conditions also reactivated expression of the EBV immediate-early lytic BZLF1 and early lytic BMRF1 proteins. By contrast, restriction of arginine, histidine, or leucine more strongly induced BZLF1 and BMRF1, particularly by day 5 of culture (Fig. 1B and C). Although histidine restriction highly induced EBV reactivation in P3HR-1, it did not induce EBV lytic antigen in two other EBV+ Burkitt cell lines (Fig. S1C), including the EB3 Burkitt lymphoma cells used by the Henles in their seminal arginine restriction study. Leucine restriction moderately reactivated BZLF1 and BMRF1 expression in two EBV+ Burkitt cell lines (Fig. S1D).

Restriction of essential amino acids, arginine, glutamine, or tyrosine also increased the percentage of annexin V+ cells somewhat, which is indicative of cell death in a subpopulation of cells (Fig. S1E). Therefore, we next examined whether the expression of EBV lytic antigens correlated with caspase activity, which can trigger EBV reactivation (62). Although restriction of multiple amino acids increased caspase activity, the magnitude of increased caspase activity did not correlate with annexin V+ positivity or with the extent of EBV reactivation (Fig. 1B and C; Fig. S1E and G). Similarly, the annexin V+ percentage did not correlate with BMRF1 expression levels. For example, isoleucine restriction caused the highest levels of cell death but did not reactivate BMRF1 expression (Fig. S1F). While amino acid restriction can inhibit the metabolism master regulator mTOR, rapamycin inhibition of mTOR itself did not induce EBV lytic antigen expression (Fig. S1H). Together, these results suggest that metabolic pathway(s) specific to each amino acid underlie maintenance of EBV latency, instead of merely amino acid restriction-induced cell death.

To next define amino acid restriction effects on EBV lytic genome replication, which is carried out in the early lytic phase by a viral DNA polymerase, we performed qPCR analysis of EBV genome copy number. Restriction of any of the essential amino acids, or of several non-essential amino acids, increased EBV genome copy number to varying degrees, which could be secondary to effects on replication of either latent or lytic EBC genomes. Of these, histidine, leucine, methionine, and arginine restriction most highly increased EBV genome copy number in comparison to levels observed in EBV+ Burkitt cells grown in replete media (Fig. 1D). Since EBV genome copy number can also differ within latency (63), to differentiate between effects on latent versus lytic genome replication, we repeated the assay in the presence of acyclovir to block lytic EBV genome amplification. Interestingly, arginine restriction increased EBV copy number even in acyclovir-treated cells (Fig. 1E and Fig. S1I). Likewise, arginine restriction increased EBV genome copy number in EBV+ Raji Burkitt cells, in whom an EBV genomic BALF2 deletion prevents lytic DNA replication (Fig. S1J) (64). Therefore, arginine restriction amplifies latent EBV genome copy number, either by provoking extra rounds of latent episome replication or by increased latent genome replication within the S-phase (65, 66).

### Arginine restriction leads to an abortive EBV lytic cycle

**Fig 2:**
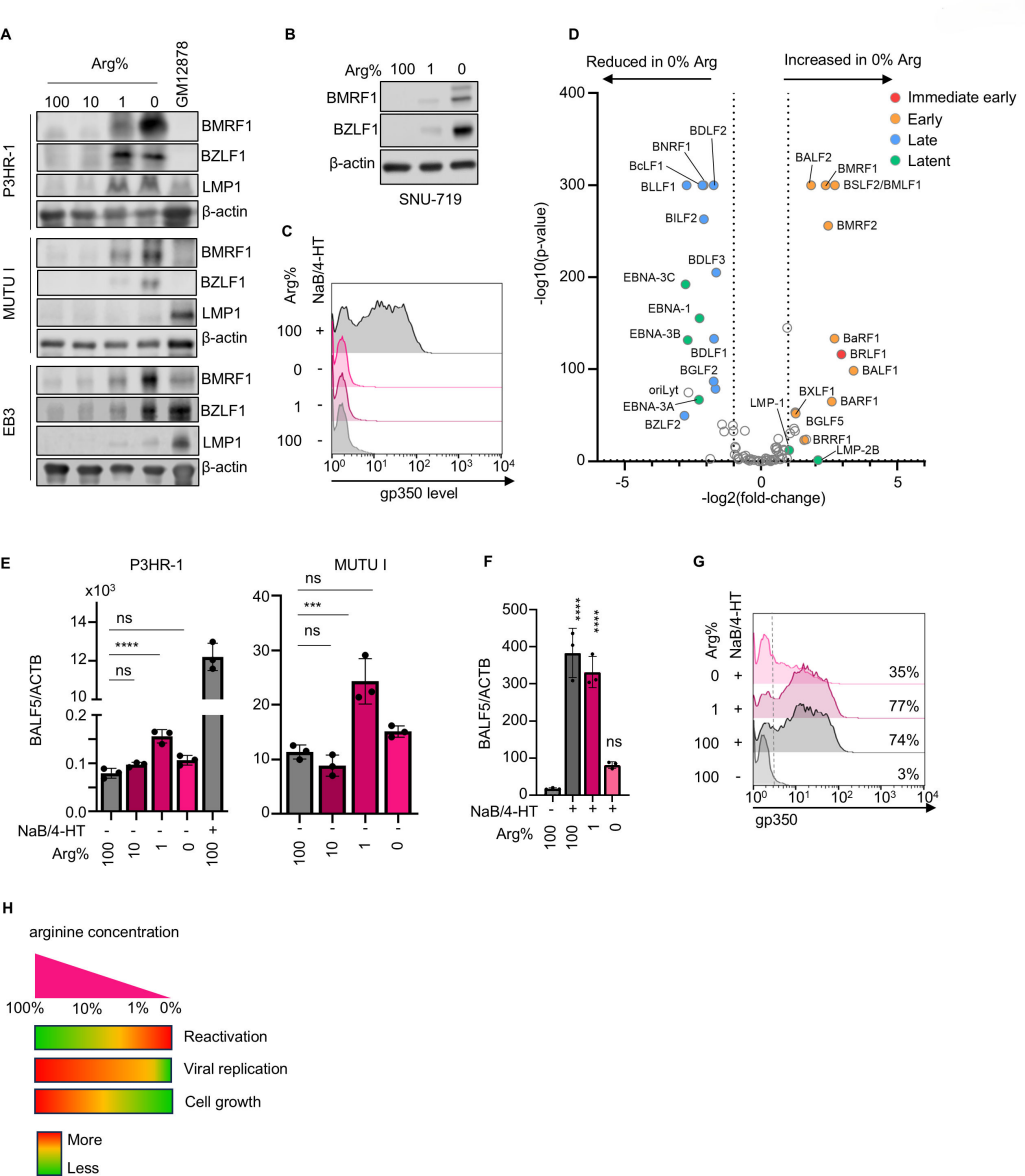
Characterization of arginine restriction effects on EBV lytic reactivation. (**A**) Analysis of arginine restriction effects on BZLF1 and LMP1 induction. Immunoblot analysis of WCL from EBV+ P3HR-1, MUTU I, or EB3 Burkitt cells cultured in media with the indicated arginine level for 5 days. WCL from GM12878 LCLs, which express the EBV latency III program, was used as a positive control for LMP1 expression. (**B**) Arginine restriction effects on EBV reactivation in SNU-719 gastric carcinoma cells. Immunoblot of WCL from SNU-719 cultured in RPMI with the indicated Arg level for 5 days. (**C**) FACS analysis of plasma membrane gp350 levels in P3HR-1 cells cultured in media with the indicated arginine concentration for 5 days. As a positive control, P3HR-1 with a conditional 4-hydroxytamoxifen (4-HT) responsive lytic reactivation system (ZHT/RHT) grown in replete media and reactivated with 4-HT (400 nM) and sodium butyrate (NaB, 0.5 mM) for 24 h are shown. (**D**) Volcano plot analysis of EBV transcriptomic responses of P3HR-1 cells cultured in media with 100% vs 0% arginine. Arginine restriction increased EBV immediate-early and early, but not late, gene expression. (**E**) qPCR analysis of intracellular EBV genome copy number in EBV+ P3HR-1 or MUTU I cells cultured in media with the indicated arginine concentration for 5 days. Shown are the ACTB-normalized mean ± SD values from *n* = 3 replicates. (**F**) qPCR analysis of EBV genome copy number in P3HR-1 ZHT/RHT cells (as in Fig. 1C) cultured in media with the indicated arginine concentrations and reactivated by 4-HT/NaB treatment for 24 h. Shown are the mean ± SD ACTB-normalized BALF5 copy number from *n* = 3 biological replicates. (**G**) FACS analysis of plasma membrane gp350 values in P3HR1 ZHT/RHT cells treated as in panel **F**. (**H**) Summary of extracellular arginine effects on EBV reactivation. Red represents stronger reactivation; green represents stronger maintenance of latency. Two-way ANOVA was used for P3HR-1 in panels **E** and **F**; one-way ANOVA was used for MUTU I in panel **E**, with ****P < 0.0001, ***P < 0.001, **P < 0.01, *P < 0.05. Blots in panels **A** and **B** are representative of *n* = 3 replicates.

We observed strong arginine restriction effects across all EBV+ Burkitt models tested (Fig. 2A) and also in EBV+ SNU-719 gastric carcinoma cells (Fig. 2B). We therefore focused on how arginine restriction reactivates EBV immediate-early and early lytic antigens. First, to define the extent of arginine restriction required for EBV reactivation, Burkitt cells were incubated in media with a range of arginine concentrations. RPMI contains 1.15 mM arginine, whereas plasma arginine typically ranges from 41 μM to 114 μM, and can be lowered with dietary arginine restriction to the 20–70mM range (67, 68). Reduction of arginine RPMI levels by 90% to 115 μM, similar to the physiological arginine level, did not alter Burkitt cell growth over 7 days (Fig. S2A). By contrast, further restriction of extracellular arginine by 99% to 11.5 μM induced growth arrest, but only low levels of cell death. Similar observations even extended to EBV+ Burkitt cells cultured in arginine-free media for 5 days (Fig. S2B). In cells cultured for 5 days at 11.5 μM (1% of typical RPMI arginine concentration), BZLF1 and BMRF1 were expressed, which was also observed in SNU-719 gastric carcinoma cells (Fig. 2A and B). BMRF1 expression was also detectable by confocal immunofluorescence microscopy analysis in cells cultured in arginine-free media for 5 days (Fig. S2C and D).

When cultured in media with 1% or 0% the levels of arginine found in RPMI for 5 days together with dialyzed fetal calf serum to remove free amino acids, LMP1 expression was also induced in P3HR-1 cells and to a lesser degree in EB3, but not in MUTU I Burkitt cells (Fig. 2A). Since LMP1 can be expressed as a latency oncogene within the latency IIa or III programs versus as a lytic gene whose expression is highest at late periods of reactivation with roles that both support and inhibit the lytic cycle (69–71), it is possible that such complex regulation accounts for differences across Burkitt lymphoma models.

We tested whether apoptotic cells account for EBV reactivation with arginine restriction. We used fluorescence-activated cell sorting (FACS) to sort annexin+ dead versus annexin V− live cells. While both annexin+ and annexin− populations expressed LMP1, annexin V-negative cells accounted for most BMRF1 and BZLF1 expression (Fig. S2E). To further assess whether there was a relationship between cell death and EBV reactivation under arginine restriction conditions, we stained cells with Zombie Aqua, a fixation-compatible cell death dye, followed by staining for BMRF1. In agreement with the annexin V analysis, live Zombie Aqua-negative cells more highly expressed BMRF1 (Fig. S2F). Finally, treatment with the pan-caspase inhibitor Z-VAD-FMK (20 μM) throughout the 5 days of arginine restriction did not block EBV lytic reactivation, despite inhibition of caspase activity (Fig. S2G and H). These data indicate that arginine restriction reactivates EBV lytic gene expression independently of apoptosis.

We next assessed whether arginine restriction triggered EBV lytic cycle DNA replication or expression of the EBV late lytic antigen gp350, which is expressed from newly synthesized lytic EBV genome templates. Arginine restriction mildly increased EBV genome copy number, though not gp350 expression (Fig. 1E and 2C). To more systematically define arginine restriction effects on EBV lytic antigen expression, we performed RNAseq analysis on EBV+ P3HR-1 grown under replete versus arginine-restricted conditions. Whereas arginine restriction reactivated expression of EBV immediate-early and early lytic mRNAs, it did not reactivate late gene expression. In fact, we observed higher late lytic gene expression in cells grown in replete media, likely reflecting rare cells with spontaneous reactivation of a complete EBV lytic cycle (Fig. 2D). These results suggest that metabolism of extracellular arginine may be necessary for EBV late lytic gene expression, likely at the stage of lytic DNA replication. Consistent with this, EBV genome copy number was significantly higher in cells cultured in 1% arginine than in arginine-free media, despite higher BZLF1 and BMRF1 expression in arginine-free media (Fig. 2E).

From above results, we therefore hypothesized that arginine might be essential for EBV lytic DNA replication, as arginine is a key building block for nucleotide synthesis and is important for herpes simplex virus replication (72). To test this hypothesis, P3HR-1 cells that express a conditional lytic reactivation system, in which tamoxifen (4-HT) triggers nuclear translocation of BZLF1 and BRLF1 immediate-early proteins (73, 74), were seeded in media with a range of arginine concentrations and induced for lytic reactivation by 4HT and the histone deacetylase inhibitor sodium butyrate (NaB). Viral DNA replication and late protein gp350 expression were only modestly decreased when cells were grown in media containing 1% of typical RPMI arginine concentration. By contrast, culture in arginine-free media strongly blocked EBV lytic DNA replication and late lytic protein gp350 expression (Fig. 2F and G). These data indicate that Burkitt cell proliferation and viral lytic DNA replication differ in their dependence on extracellular arginine concentration (Fig. 2H), and that severe arginine restriction resulted in abortive EBV reactivation, where immediate-early and early genes are reactivated, but viral lytic genomes are not synthesized and consequently late genes are not expressed.

### Metabolomic analysis of arginine-restricted EBV+ Burkitt cells

**Fig 3:**
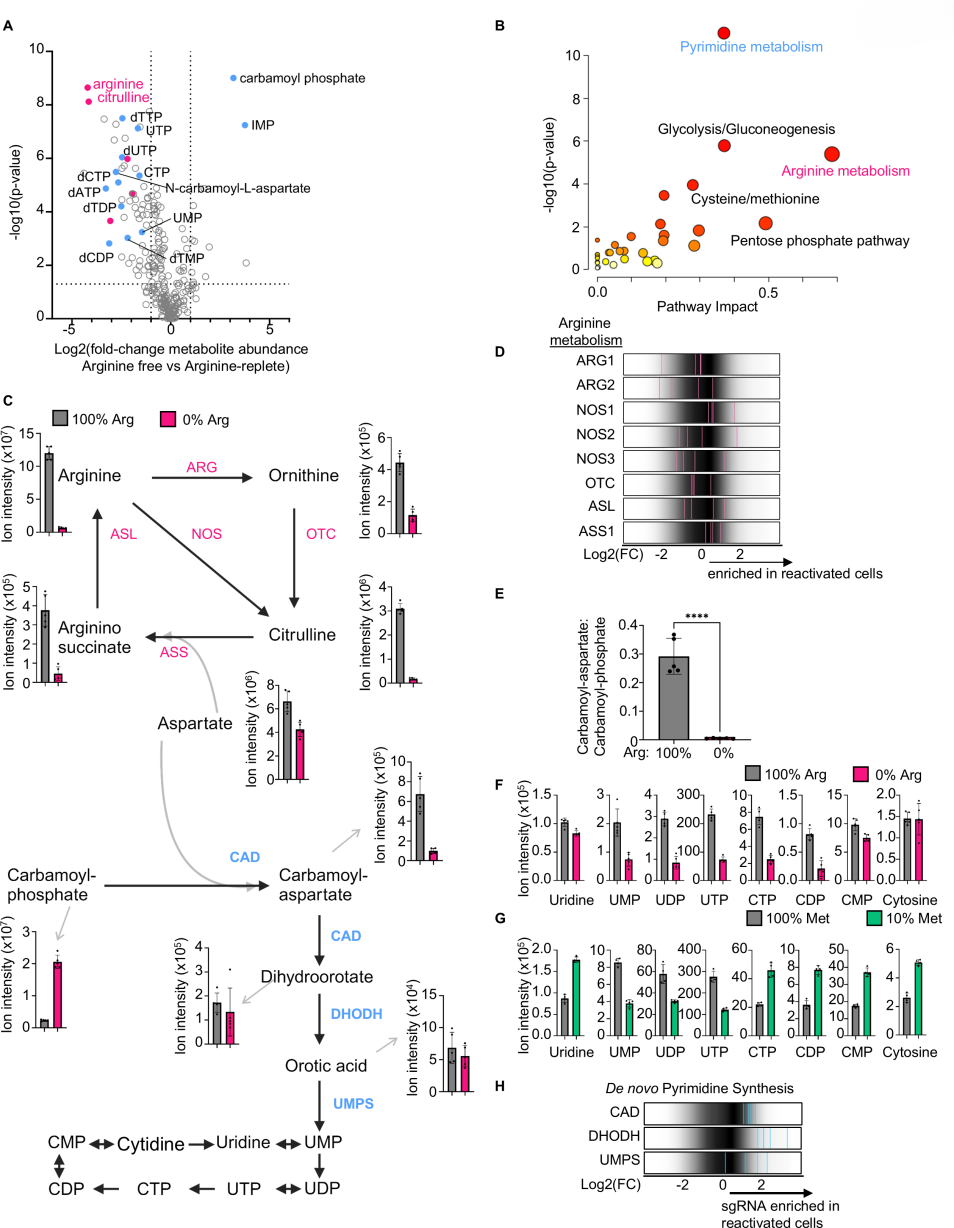
Arginine restriction impairs EBV+ Burkitt d*e novo* pyrimidine biosynthesis (**A**) Analysis of arginine restriction effects on the Burkitt B-cell metabolome. Volcano plot of LC-MS metabolomic analysis of EB3 Burkitt cells cultured arginine-free vs arginine-replete media for 5 days, from *n* = 5 replicates. Higher fold change values indicate higher levels in cells grown under arginine-free conditions. Arginine cycle metabolites are highlighted in pink; pyrimidine- and purine-related metabolites are highlighted in blue. (**B**) KEGG metabolic pathway analysis of LC-MS data as in panel **A**. Metabolites were selected by FDR < 0.05, and pathway impact values were computed by MetaboAnalyst 3.0 topological analysis. (**C**) Schematic of arginine cycle and *de novo* pyrimidine synthesis pathways. Shown at right are abundances of indicated metabolites in EB3 cells grown under arginine-replete (100% Arg) versus arginine-free (0% Arg). (**D**) Rug plots of sgRNA levels targeting arginine metabolism genes from a human genome-wide CRISPR screen for host factors that maintain EBV latency in P3HR-1 cells (75). Shown are the log_2_-transformed fold change abundances in gp350+ sorted P3HR-1 versus in the input library, where the magenta lines represent values for each of the four independent sgRNA targeting the specified gene. The overall distribution of human genome-wide Brunello library sgRNAs is shown in gray in the background for reference, where most sgRNAs did not perturb EBV latency. Higher values indicate sgRNA enrichment in reactivated gp350+ cells versus in the input library as a whole. (**E**) CAD activity, as read out by the ratio of the CAD product carbamoyl aspartate to the CAD precursor carbamoyl phosphate in EB3 cells, as in panel **A**. *P*-values were calculated by Student’s *t*-test. ****P < 0.0001. (**F**) Pyrimidine abundance from EB3 cells as in panel **A**. (**G**) Abundance of pyrimidine metabolites from P3HR-1 cells cultured in 10% methionine vs 100% methionine media for 72 h (76). (**H**) Rug plots highlighting fold change abundances of the indicated *de novo* pyrimidine synthesis gene-targeting sgRNAs in gp350+ cells shown in teal (four independent sgRNAs per gene) in a human genome-wide CRISPR screen for P3HR-1 factors that maintain EBV latency, as in panel **D** (75).

To further investigate metabolic signals and pathways that underlie arginine restriction-driven EBV reactivation, we performed liquid chromatography-mass spectrometry (LC-MS) analyses of polar metabolites, using extracts from EB3 Burkitt cells incubated in replete RPMI/FCS versus arginine-free RPMI/FCS for 5 days. As expected, arginine itself, and several closely related arginine metabolites, including ornithine, citrulline, and argininosuccinate, were highly depleted in cells grown in arginine-free media. Interestingly, multiple nucleosides and nucleotide precursors were also significantly depleted by arginine restriction, while the pyrimidine precursor carbamoyl phosphate was instead more abundant in cells grown in arginine-free media (Fig. 3A).

Metabolomic pathway analysis further highlighted that arginine and pyrimidine metabolism were among the most significantly altered processes in cells grown under arginine-free conditions (Fig. 3B). To a lesser degree, glycolysis and gluconeogenesis pathways were also altered, consistent with prior reports that arginine starvation causes respiratory stress and inhibits aerobic glycolysis in transformed cells (47). While ATP levels were lower in cells grown under arginine-free conditions, ADP/ATP ratios were similar in cells grown under arginine-replete vs arginine-free conditions, suggesting that arginine restriction does not block energy production *per se* (Fig. S3A and B).

We explored whether arginine restriction caused secondary effects on the levels of other amino acids whose restriction drove EBV reactivation. Interestingly, arginine restriction did not significantly alter levels of glutamine, histidine, leucine, or tryptophan (Fig. S3C). Similarly, LC-MS metabolomic analyses of cell culture media following Burkitt culture for 3 or 5 days in replete versus arginine-free media indicated that arginine restriction did not significantly alter levels of any other amino acid (Fig. S3D). As expected, arginine restriction decreased levels of arginine metabolites ornithine and citrulline.

The rate-limiting *de novo* arginine biosynthesis enzyme ASS1 combines citrulline and aspartate to produce argininosuccinate, which is then used by argininosuccinate lyase to regenerate arginine (Fig. 3C). Notably, arginine restriction increased argininosuccinic acid levels on day 3 of culture (Fig. S3D), likely as cells attempted to compensate for the loss of arginine uptake. Lymphomas frequently silence ASS1 expression (77), which can be leveraged by arginine restriction therapeutic approaches in pre-clinical development to trigger oxidative stress and mitochondrial dysfunction (47, 49). We therefore surveyed ASS1 expression in EBV-infected Burkitt and gastric carcinoma cell contexts. Arginine restriction increased ASS1 expression in EBV+ EB3 Burkitt cells and in EBV+ SNU-719 gastric carcinoma cells, but only moderately in two other EBV+ Burkitt cell lines (Fig. S3E), which had variable ASS1 expression.

We next examined if arginine restriction-driven changes in arginine metabolite abundance might trigger EBV reactivation. Intracellular arginine can be catabolized by nitric oxide (NO) synthases (NOS), arginine:glycine amidinotransferase, arginase (ARG), or by arginine decarboxylase to produce NO/citrulline, guanidinoacetate, ornithine, and agmatine, respectively (Fig. 3C). Ornithine can be further metabolized to generate citrulline via ornithine transcarbamylase (OTC). Since ornithine and citrulline were both significantly decreased by Burkitt cell culture in arginine-free media (Fig. 3C), we investigated whether depletion of ARG, OTC, or NOS altered EBV reactivation. To gauge the involvement of these in EBV latency maintenance, we examined our lab’s recent human genome-wide CRISPR-Cas9 screen for host factors that maintain Burkitt EBV latency (75). CRISPR single-guide RNAs (sgRNAs) targeting arginine metabolism genes, including ARG, NOS, or OTC, were not enriched in cells triggered for EBV lytic reactivation by CRISPR editing, arguing against their obligatory roles in maintenance of EBV latency (Fig. 3D). Consistent with this, supplementation of 1.15 mM ornithine or citrulline (the RPMI arginine molarity) did not reduce EBV lytic gene expression in three EBV+ Burkitt cells cultured in arginine-free media (Fig. S3F). Interestingly, supplementation of ornithine and citrulline did, however, reduce BZLF1 and BMRF1 expression in SNU-719 gastric carcinoma cells (Fig. S3G), highlighting potentially distinct arginine metabolism dependencies in EBV+ B versus epithelial cell contexts.

We then examined changes in pyrimidine biosynthesis pathways in arginine-restricted cells. *De novo* pyrimidine biosynthesis requires the trifunctional enzyme CAD, which first uses its aspartate transcarbamoylase domain to synthesize carbamoyl aspartate from carbamoyl phosphate and aspartate (Fig. 3C). The CAD dihydroorotase domain then metabolizes carbamoyl aspartate to dihydroorotate, which is converted to UMP by the enzymes DHODH and UMPS (57) (Fig. 3C). Metabolomic analysis highlighted that Burkitt cell CAD carbamoyl-phosphate synthetase activity, defined by the carbamoyl-phosphate to carbamoyl-aspartate precursor/product ratio, was strongly inhibited by arginine restriction. This correlated with decreased pyrimidine levels, including UTP and CTP (Fig. 3E and F). By contrast, Burkitt methionine restriction (76) instead increased CTP levels (Fig. 3G). Intriguingly, CRISPR screen analysis suggests that perturbation of the *de novo* pyrimidine pathway by knockout of CAD, DHODH, or UMPS triggers EBV reactivation, as sgRNAs targeting each of these were highly enriched in the population of CRISPR-reactivated Burkitt cells (Fig. 3H). Taken together, these data suggest that *de novo* pyrimidine synthesis and likely therefore also abundant pyrimidine supply support maintenance of EBV latency.

### *De novo* pyrimidine biosynthesis is necessary for Burkitt B-cell EBV latency

**Fig 4:**
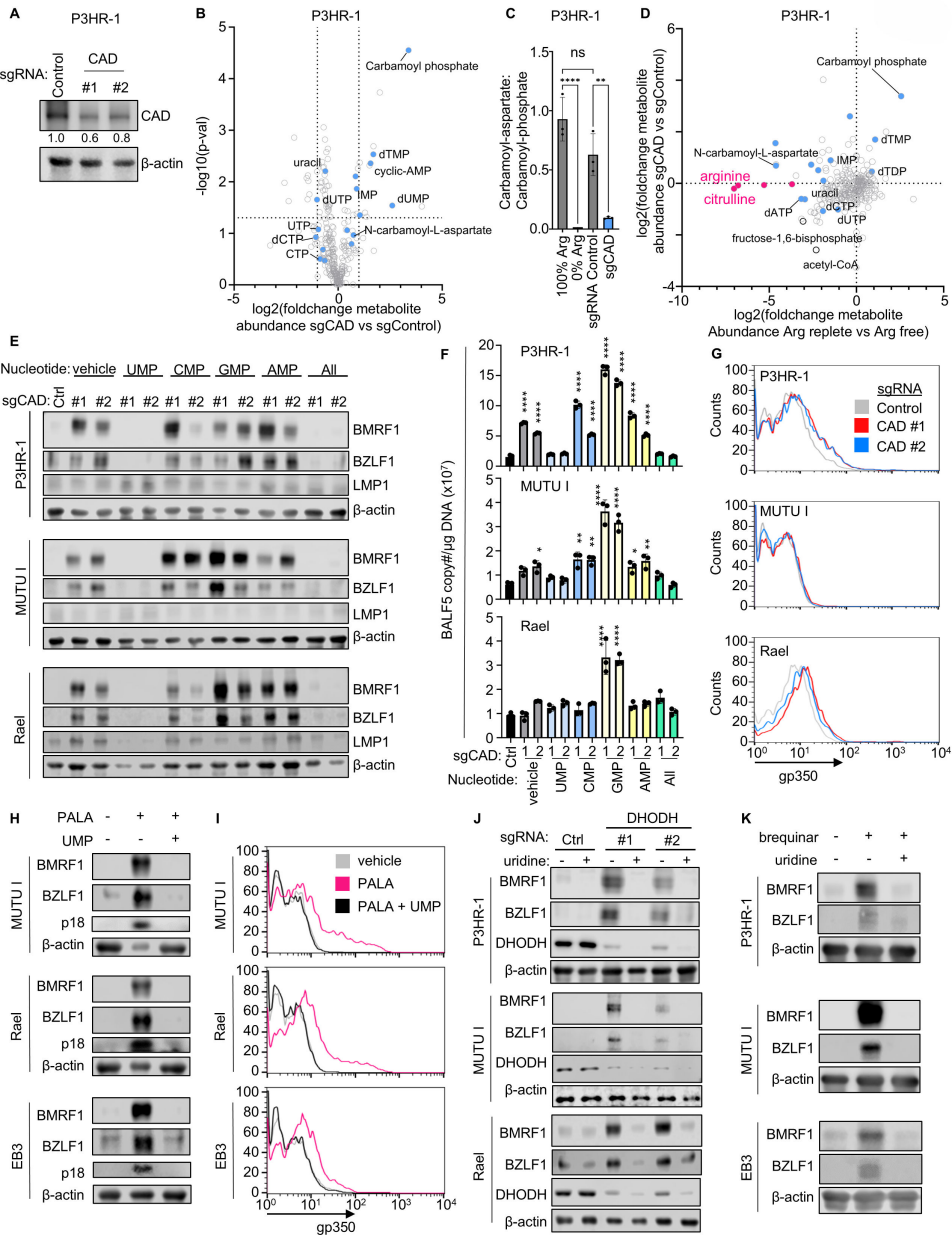
*De novo* pyrimidine biosynthesis is necessary for maintenance of Burkitt EBV latency. (**A**) Immunoblot analysis of WCL from P3HR-1 cells expressing the indicated control or CAD-targeting sgRNAs for 6 days. (**B**) Volcano plot of LC-MS metabolomic analysis of P3HR-1 cells expressing control or CAD-targeting sgRNAs for 6 days from *n* = 3 replicates. Shown are the metabolite fold change levels from CAD-depleted versus control cells. Higher fold change indicates increased levels in CAD-depleted cells. Nucleotide-related metabolites are highlighted in blue. (**C**) CAD activity, as judged by the carbamoyl aspartate/carbamoyl phosphate ratio, in P3HR-1 cultured in arginine-free vs replete RPMI media for 5 days, or expressing CAD vs control sgRNAs. (**D**) Volcano plot of log_2_ fold-change metabolite abundance from metabolomic analysis of P3HR-1 cells that were cultured in arginine-free vs replete media as in panel **B** (*x-*axis) versus in CAD-depleted vs control P3HR-1 (*y*-axis). Arginine cycle metabolites are highlighted in pink; pyrimidine- and purine-related metabolites are highlighted in blue. (**E**) Analysis of nucleotide rescue effects on EBV latency in CAD-depleted cells. Immunoblot analysis of WCL from cells that expressed control or CAD-targeting sgRNA and that were cultured in media supplemented with the indicated nucleotides at 50 μg/mL, added from 48 h after sgRNA expression. (**F**) Analysis of nucleotide rescue effects on EBV lytic genome replication in CAD-depleted cells. Shown are mean ± SD values from qPCR analysis of intracellular EBV genome copy number from cells as in panel **E**. (**G**) FACS analysis of plasma membrane gp350 level in the indicated Burkitt cells that expressed control or CAD-targeting sgRNA. (**H**) Immunoblot analysis of WCL from Burkitt cells cultured in RPMI media containing the CAD inhibitor PALA (250 μM) and/or UMP (50 μg/mL) as indicated for 4 days. (**I**) FACS analysis of plasma membrane gp350 expression in cells cultured as in panel **H**. (**J**) Immunoblot analysis of WCL from cells that expressed control or DHODH-targeting sgRNA and cultured in RPMI supplemented with 50 μg/mL uridine, as indicated. (**K**) Immunoblot analysis of WCL from cells cultured with the DHODH inhibitor brequinar (BQR; 3 μM) and with uridine (50 μg/mL) for 4 days, as indicated. Two-way ANOVA was performed for panels **C** and **F**. **** *P* < 0.0001, ***P < 0.001, **P < 0.01, *P < 0.05. Blots are representative of *n* = 3 replicates.

We next tested whether *de novo* pyrimidine biosynthesis was necessary for maintenance of EBV latency within Burkitt B cells. First, we used CRISPR to deplete CAD within P3HR-1 Burkitt cells (Fig. 4A). We then performed LC-MS metabolomic analysis to define CAD KO effects on metabolite abundances. Suggestive of on-target CRISPR effects, the CAD precursor carbamoyl phosphate was the most highly increased metabolite by CAD depletion. Similar to arginine restriction, CAD depletion highly altered levels of multiple nucleotides and nucleotide-related metabolites, with UTP among the most decreased (Fig. 4B). Similarly, shifting P3HR-1 from arginine-replete to arginine-free media for 5 days strongly decreased the ratio of CAD product carbamoyl aspartate to CAD precursor carbamoyl phosphate and perturbed levels of multiple nucleotides (Fig. 4C and D). Arginine restriction also highly depleted arginine and the arginine metabolites citrulline and ornithine. Metabolomic pathway impact analysis highlighted that arginine restriction most highly perturbed arginine biosynthesis, as expected, but also highly perturbed purine and pyrimidine metabolism (Fig. 4D; Fig. S4A and B). Arginine restriction similarly altered metabolites in EBV+ P3HR-1 and EB3 Burkitt cells, in both of whom arginine, citrulline, and ornithine levels were strongly diminished (Fig. S4C). Interestingly, arginine restriction, but not CAD KO, strongly decreased intracellular arginine and citrulline levels (Fig. 4D). Arginine restriction and CAD KO each depleted ATP, CTP, and UTP levels, but increased TMP and TDP levels (Fig. 4D).

We next tested whether *de novo* synthesis of pyrimidine biosynthesis was necessary for EBV latency. In support of this hypothesis, CAD KO-induced BZLF1 and BMRF1 expression, which were fully suppressed by UMP supplementation (Fig. 4E). UMP can be metabolized to all other pyrimidines, but supplementation with the nucleotides cytidine monophosphate (CMP), guanosine monophosphate (GMP), or adenosine monophosphate (AMP) failed to suppress arginine restriction-driven EBV reactivation (Fig. 4E). Interestingly, CAD KO increased intracellular EBV genome copy number, and this could also be suppressed by UMP but not by CMP, GMP, or AMP supplementation (Fig. 4F). EBV DNA FISH analysis highlighted that CAD KO strongly induced EBV genome amplification in a subset of cells, and importantly, this signal was blocked by acyclovir in both P3HR-1 and Mutu I, indicating that CAD KO-induced lytic EBV genome amplification (Fig. S4D and E). We did not observe differences in FISH-defined puncta indicative of latent EBV genomes in cells without this bright FISH signal or between control and acyclovir-treated CAD KO cells (Fig. S4D and F), suggesting that the short duration of CAD KO did not select for cells with altered latent EBV genome copy number.

Acyclovir blocked CAD KO-induced EBV genome copy number amplification (Fig. S4G), further indicating that CAD activity is required for maintenance of EBV latency. Interestingly, however, GMP supplementation significantly boosted EBV genome copy number in CAD KO cells (Fig. 4F). GMP supplementation also increased EBV genome copy number in Burkitt cells grown in replete media, but did not induce EBV lytic antigens, suggesting an effect at the level of latent genome replication (Fig. S5A and B). Although UMP supplementation did not maintain EBV latency in arginine-restricted cells (Fig. S5C), uridine or cytidine supplementation somewhat blocked BZLF1 and BMRF1 expression in P3HR-1 or MUTU I cells grown in arginine-free media (Fig. S5D). CAD KO also modestly increased gp350 expression in EBV+ P3HR-1 and Rael cells (Fig. 4G). Taken together, these results highlight key CAD and *de novo* pyrimidine biosynthesis roles in control of EBV genome copy number and lytic reactivation.

We next treated EBV+ Burkitt cells with the CAD aspartate transcarbamylase inhibitor PALA (N-phosphonacetyl-L-aspartate) (78) to block the first three steps of *de novo* pyrimidine biosynthesis, which produces dihydroorotate from glutamine and aspartate. PALA robustly induced EBV early lytic antigen expression, validating our CAD KO CRISPR analyses. In contrast to arginine restriction but consistent with CAD KO, PALA increased EBV late lytic p18 capsid and gp350 protein expression in multiple Burkitt cell contexts. These were on-target effects because each was rescuable by UMP supplementation (Fig. 4H and I; Fig. S5E and F). Furthermore, acyclovir blocked EBV genome copy number increase and gp350 expression in PALA-treated cells (Fig. S5G and H). Interestingly, however, arginine restriction blunted the extent to which PALA reactivated BZLF1 and BMRF1 expression (Fig. S5I). These results are consistent with a model in which nucleotide imbalance drives EBV lytic reactivation, or alternatively that EBV latency requires arginine import to support uridine uptake and/or uridine salvage pathway metabolism (79).

We next tested whether the mitochondrial enzyme DHODH, which converts dihydroorotate and oxidized quinone to orotate and reduced quinone, and which can form a “pyrimidinosome” complex together with CAD and UMPS across mitochondrial membranes (57), was necessary for EBV latency. We perturbed EBV+ Burkitt DHODH activity by CRISPR editing or by the small molecule DHODH inhibitor brequinar (80, 81). DHODH inhibition induced BZLF1 and BMRF1, which could be rescued by uridine supplementation, suggestive of on-target effects on the *de novo* pyrimidine biosynthesis pathway (Fig. 4J and K). These results suggest that DHODH orotate synthetase activity, and not quinone reductase activity, is critical for EBV latency.

### Arginine restriction triggers DNA damage to reactivate EBV

To gain further insights into how arginine restriction triggers EBV reactivation, we measured changes in host gene expression in EBV+ Burkitt cells grown under replete versus arginine restriction conditions by RNAseq. While arginine restriction had only moderate effects on the host transcriptome (Fig. S6A), “EBV infection response” was among the most highly upregulated KEGG pathways, whereas pyrimidine and pentose phosphate pathways were among the most downregulated (Fig. S6B). DNA damage repair pathways were also highly downregulated by arginine restriction, whereas p53 signaling was increased (Fig. S6B).

**Fig 5:**
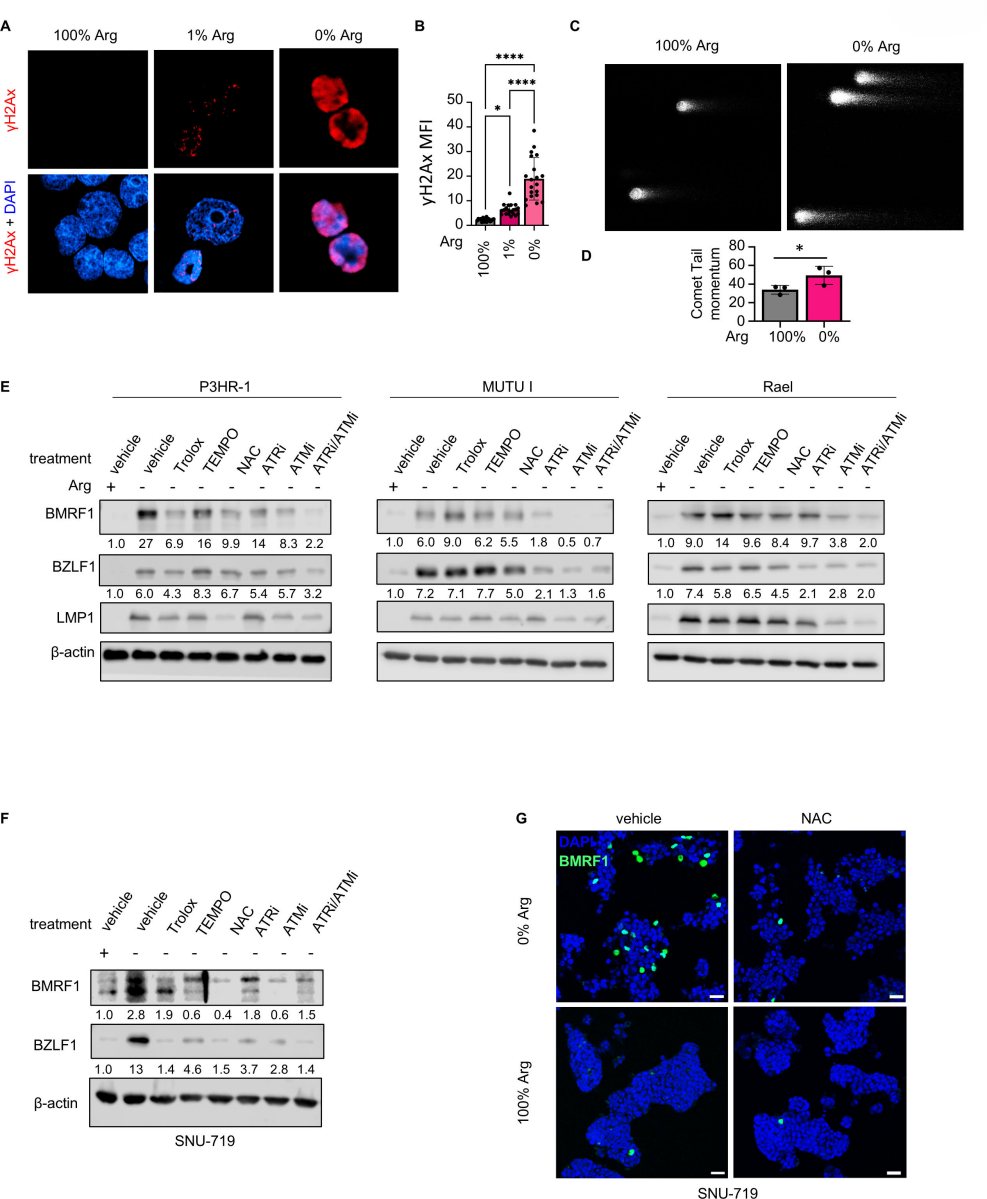
DNA damage responses contribute to EBV arginine restriction-driven EBV reactivation. (**A**) Analysis of arginine restriction effects on Burkitt B-cell DNA damage. Representative confocal microscopy analysis of DNA damage marker γH2Ax in P3HR-1 cells cultured in RPMI with the indicated arginine levels for 5 days. (**B**) Mean ± SD of γH2Ax MFI in cells as in panel **A**. Twenty randomly chosen cells from *n* = 3 replicates were quantified. (**C**) Comet assay analysis of P3HR-1 cells cultured in replete or arginine-free media for 5 days. (**D**) Mean ± SD comet assay tail moment from *n* = 3 replicates as in panel **C**. *P < 0.05, paired *t*-test. (**E**) Effects of antioxidants or DNA damage inhibitors on arginine restriction-driven EBV reactivation. Immunoblot analysis of WCL from the indicated cells cultured in replete or arginine-free media, with the antioxidants Trolox (50 μM), TEMPO (12.5 μM), NAC (10 mM) and/or with ATR inhibitor VE-822 (ATRi, 5 μM), or ATM inhibitor KU-60019 (ATMi, 5 μM) for 5 days. (**F**) Immunoblot analysis of WCL from EBV+ SNU-719 gastric carcinoma cells treated as in panel **E**. (**G**) Representative confocal immunofluorescence images of BMRF1 expression (green) versus nuclear DAPI stain in SNU-719 cells grown in arginine-replete or -free media for 5 days, in the absence or presence of 10 mM NAC as in panel **E**. Scale bars = 50 µm. Blots are representative of *n* = 3 replicates.

Taken together with the above results, we therefore hypothesized that arginine restriction triggers EBV reactivation through effects on nucleotide pool imbalance and DNA damage. Consistent with this hypothesis, arginine restriction increased γH2Ax signals, a marker of DNA damage in P3HR-1 cells (Fig. 5A and B). To examine this further, we next used immunoblotting to measure phosphorylation of the kinases ATM (ataxia-telangiectasia mutated) and ATR (ATM- and Rad3-related), which are activated by double-strand or single-strand DNA breaks, respectively, as a readout of their activity. ATR can also be activated by replication stress (82–84). Arginine restriction induced ATM phosphorylation or ATM and ATR phosphorylation in multiple EBV+ Burkitt cell lines (Fig. S6C). Also indicative of increased DNA breakage, arginine restriction also increased comet assay tail moment (85) (Fig. 5C and D).

Since BZLF1 expression alone can induce DNA damage (86), we also examined DNA damage responses to arginine restriction in EBV-negative Burkitt cells. Interestingly, arginine restriction induced phosphorylation of ATR in EBV− MUTU cells (Fig. S6D), suggesting that EBV reactivation is not required for arginine restriction-driven Burkitt B-cell DNA damage. To test whether DNA damage responses were required for arginine restriction-induced EBV reactivation, we evaluated effects of the ATM inhibitor VE-822, the ATR inhibitor KU-60019, or both. Arginine restriction-driven EBV reactivation was partially blocked by either ATM or ATR inhibition, and combined ATR/ATM inhibition had additive effects on EBV reactivation expression (Fig. 5E). These results suggest that the ATM and ATR pathways may each serve as drivers of EBV reactivation downstream of arginine restriction, which we note frequently accumulate mutations in DNA damage pathways (87).

Disruption of mitochondrial membrane potential inhibits *de novo* pyrimidine biosynthesis at the level of DHODH and activates downstream p53 signaling, in part through alteration of reactive oxygen species (ROS) levels (59). To explore potential ROS roles downstream of arginine restriction, we treated EBV+ Burkitt or SNU-719 gastric carcinoma cells with vehicle control or with the antioxidants NAC (N-acetylcysteine, 10 mM), Trolox (50 μM), or TEMPO (125 μM) for 5 days, together with arginine restriction. NAC, Trolox, or TEMPO treatment had modest to no effects on P3HR-1, MUTU I, or Rael Burkitt cell reactivation (Fig. 5E). Interestingly, however, they did impair arginine restriction-driven EBV lytic reactivation in SNU-719 gastric carcinoma (Fig. 5F and G).

While arginine restriction did not increase total cellular ROS or mitochondrial ROS levels in EBV+ Burkitt cells, it did increase total ROS levels in SNU-719, which were suppressed by antioxidant treatment (Fig. S6E through G). Since arginine metabolites ornithine and citrulline also partially suppressed BZLF1 and BMRF1 expression in arginine-restricted SNU-719 cells, our results suggest that arginine restriction-driven ROS production may contribute to EBV reactivation in epithelial cell but not Burkitt B-cell contexts.

We tested whether DNA damage might be a final common pathway for EBV lytic reactivation by amino acid restriction. However, ATM or ATR inhibition did not reduce BZLF1 and only moderately reduced BMRF1 expression in P3HR-1 grown in leucine-free media for 5 days. By contrast, pan-caspase inhibition by Z-VAD-FMK more strongly blocked leucine restriction-driven EBV reactivation (Fig. S6H). Taken together, these results suggest that distinct cellular pathways drive EBV reactivation downstream of arginine versus leucine reactivation.

### Arginine restriction and DNA hypomethylation additively induce EBV lytic antigen expression

**Fig 6:**
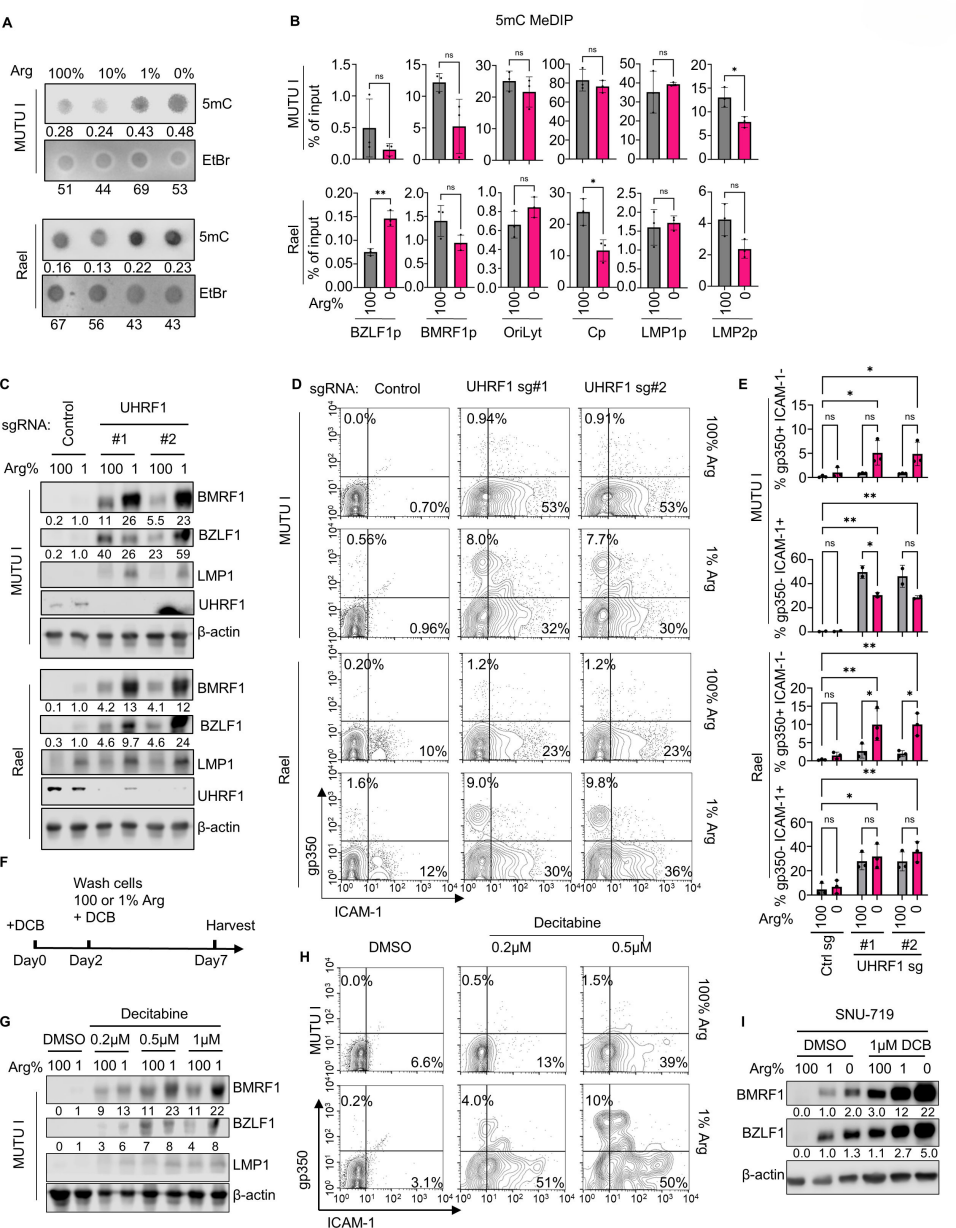
Effects of combinatorial arginine restriction and DNA hypomethylation on EBV reactivation. (**A**) Analysis of arginine restriction effects on EBV+ Burkitt 5-methylcytosine (5mC) levels. 5mC dot blot analysis of DNA extracted from MUTU I or Rael cells that were cultured in RPMI with the indicated arginine levels for 5 days. As a load control, membranes were also stained with ethidium bromide (EtBr). Each dot contains 500 ng (MUTU I) or 1 μg (Rael) of DNA. (**B**) 5mC methylated DNA immunoprecipitation-qPCR (MeDIP-qPCR) analysis of chromatin from MUTU I or Rael cells cultured in arginine-replete vs -free media for 5 days. One hundred micrograms per milliliter of acyclovir was added to prevent lytic DNA replication. Mean ± SD values from *n* = 3 replicates are shown. (**C**) Immunoblot analysis of WCL from MUTU I or Rael cells that expressed control or *UHRF1* targeting sgRNA and cultured in media with 1% or 100% of RPMI arginine levels. Cells were induced to express sgRNA for 3 days and then cultured in indicated media for 5 days. (**D**) FACS analysis of plasma membrane gp350 expression as a marker of lytic reactivation vs ICAM-1 expression as a marker of LMP1 expression in cells cultured as in panel **C**. (**E**) Mean ± SEM percentages of gp350+ or ICAM-1+ cells from *n* = 3 experiments as in panel **D**. (**F**) Schematic of arginine restriction and decitabine (DCB) treatments. (**G**) Immunoblot analysis of WCL from MUTU I cells that were cultured in media containing the indicated DCB concentrations for 2 days and then cultured in arginine restricted (1% RPMI levels) or replete media for 5 days. (**H**) FACS analysis of plasma membrane gp350 or ICAM-1 expression on cells cultured as in panel **G**. (**I**) Immunoblot analysis of WCL from SNU-719 gastric carcinoma cells that were cultured in media containing the indicated DCB concentrations for 2 days and then cultured in arginine restricted (1% Arg) or replete media for 5 days. Student’s *t*-test was performed for panel **B** and two-way ANOVA was performed for panel **E**. ****P < 0.0001, ***P < 0.001, **P < 0.01, *P < 0.05. Blots are representative of *n* = 3 replicates.

Many EBV-infected tumors, including EBV+ Burkitt lymphoma, exhibit CpG island DNA hypermethylation, and DNA hypomethylation also triggers EBV reactivation (13, 76, 88–95). We therefore examined arginine restriction effects on DNA methylation and found that withdrawal of extracellular arginine rapidly increased global Burkitt DNA methylation levels (Fig. 6A). Yet, methylated DNA immunoprecipitation-qPCR analysis revealed that arginine restriction had variable effects on EBV genomic promoter methylation levels. In P3HR-1, multiple lytic promoters exhibited decreased methylation following 5 days of culture in arginine-free media, even in the presence of acyclovir to block lytic cycle synthesis of unmethylated EBV genomes, while in the other two EBV+ Burkitt cells, the methylation status of most lytic and latency promoters did not change significantly (Fig. 6B; Fig. S7A and B). These results suggest that arginine metabolism differentially regulates host versus EBV genomic DNA methylation and raised the question of whether arginine restriction and DNA hypomethylation might synergistically or additively trigger EBV reactivation.

CpG DNA methylation marks are propagated onto newly synthesized Burkitt B-cell host and EBV genomes by the host cell enzymes DNMT1 and UHRF1 (88). We therefore next tested the effects of UHRF1 depletion on EBV reactivation in P3HR-1, MUTU I, and Rael cells, alone or together with arginine restriction for 5 days. Interestingly, UHRF1 depletion and arginine restriction more highly induced MUTU I and Rael lytic antigen expression than either did alone, suggestive of additive or synergistic effects on EBV reactivation (Fig. 6C).

We reported that DNA hypomethylation resulted in distinct populations of Burkitt cells with EBV expression (gp350+) versus with induction of the latency III program, in which LMP1 upregulated plasma membrane ICAM-1 expression (88, 96). In MUTU I and Rael cells, UHRF1 knockout and arginine restriction together additively upregulated the percentage of gp350+/ICAM-1− cells, indicative of reactivation and potentially full lytic replication, given late lytic protein expression (Fig. 6D and E). We note that this analysis may have underestimated the percentage of cells with abortive lytic EBV reactivation. UHRF1 KO and arginine restriction also significantly increased the percentage of gp350−/ICAM-1+ cells, suggestive of LMP1 induction (Fig. 6D and E), potentially with a switch to latency III. Additive gp350 upregulation was not observed in P3HR-1 cells, likely because arginine restriction alone strongly hypomethylated viral genome promoters (Fig. S7C through E). The hypomethylating agent decitabine and arginine restriction also showed additive effects on the induction of BZLF1, BMRF1, and even late gp350 expression (Fig. 6F through H; Fig. S7F and G), including in SNU-719 gastric carcinoma cells (Fig. 6I). By contrast, leucine restriction did not exhibit additive effects on EBV reactivation with decitabine treatment (Fig. S7H and I). Taken together, these data indicate that arginine restriction and DNA hypomethylation each strongly support the maintenance of the latency I program and that targeting them concomitantly enhances the switch to lytic and latency III programs within distinct single-cell populations.

**Fig 7:**
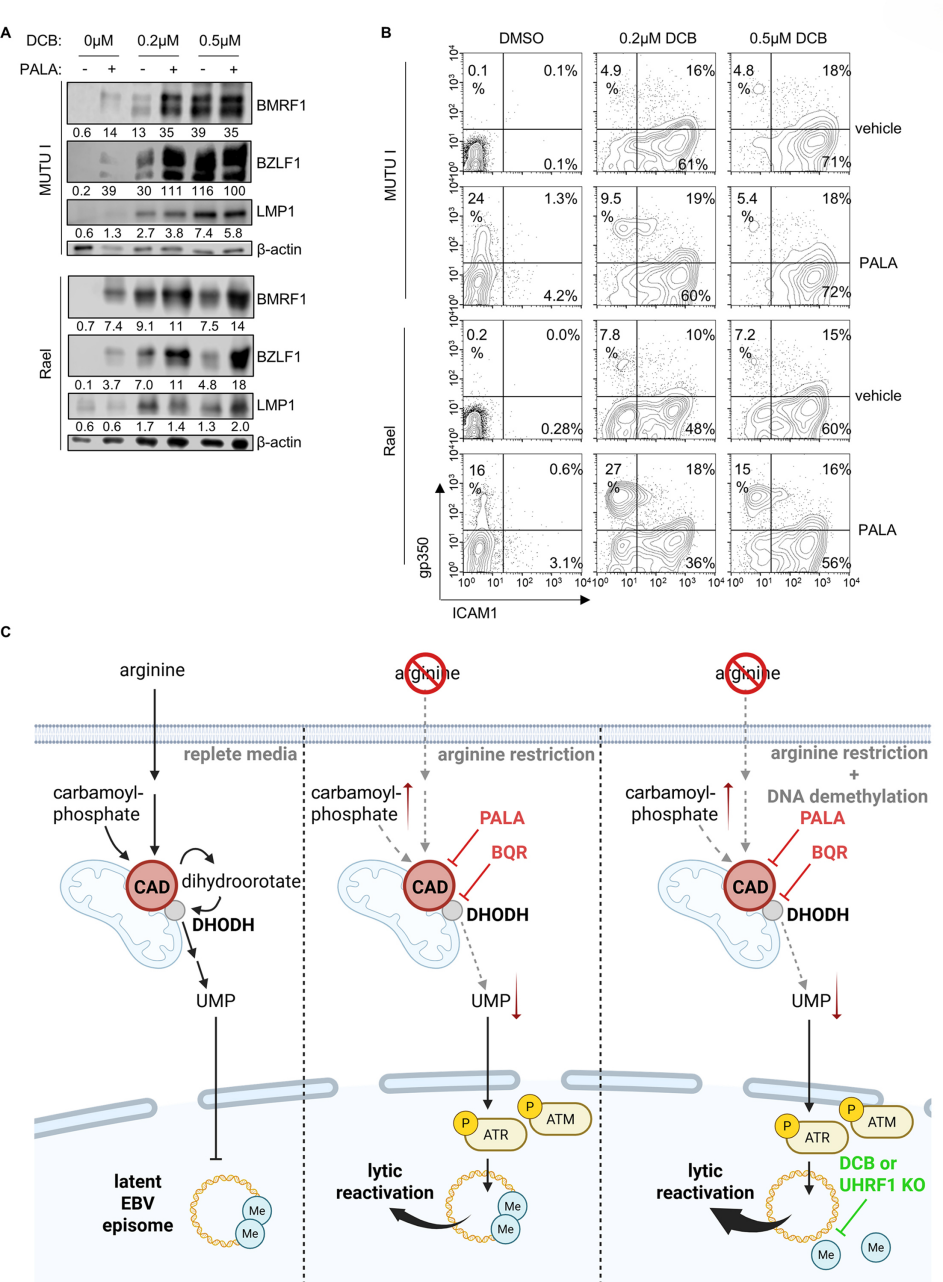
PALA and DCB additively induce EBV lytic antigen expression. (**A**) Immunoblot analysis of WCL from MUTU I or Rael cells that were treated with the indicated DCB concentrations for 2 days and then also treated with vehicle or PALA (250 μM) for 4 days, as indicated. (**B**) FACS analysis of cells treated as in panel **A**. (**C**) Schematic model of arginine and *de novo* pyrimidine metabolism effects on EBV latency, alone or together with DNA hypomethylation.

To further test potential therapeutic combinations, we further defined effects of concomitant CAD inhibition and DNA hypomethylation. MUTU I and Rael cells were treated with decitabine for 2 days and then with PALA for 4 days. At the 0.2 μM decitabine dose, PALA strongly increased EBV lytic protein expression in MUTU I and Rael (Fig. 7A). FACS analysis indicated that 0.2 μM decitabine/PALA co-treatment together significantly increased the %gp350+ cells, particularly in Rael cells (Fig. 7B), again suggesting a full lytic cycle induced by PALA and increased by decitabine co-administration. Taken together, our results support a model in which arginine metabolism supports the maintenance of the EBV latency I state through feeding the *de novo* pyrimidine synthesis pathway, whose production of UMP prevents DNA damage. Interruption of either arginine metabolism or UMP biosynthesis triggers EBV reactivation, more strongly when applied together with DNA hypomethylation (Fig. 7C).

## DISCUSSION

EBV senses the infected cell microenvironment and physiological state to dictate whether to remain latent or to reactivate, which enables viral spread but risks immune detection. Although they are a major regulator of cells’ nutritional status, much has remained unknown about how cross-talk between amino acid metabolic pathways and the EBV epigenome regulates the viral lytic switch. Here, we build on a classic observation by founders of the EBV field to investigate the mechanism by which arginine metabolism supports EBV latency within Burkitt B cells and other EBV-infected cell contexts. Metabolomic profiling highlighted that arginine metabolism supports *de novo* pyrimidine synthesis, which was necessary to support infected cell levels of uridine monophosphate, the key precursor for all pyrimidine nucleotides. Restriction of extracellular arginine or inhibition of the rate-limiting *de novo* pyrimidine biosynthesis enzyme CAD, which is supplied by upstream arginine metabolism, activated ATM- and ATR-driven DNA damage signaling. Arginine restriction and DNA hypomethylation together more highly induced EBV latency I than either alone, suggesting novel therapeutic approaches.

We note that restriction of either methionine (76) or arginine induced EBV immediate-early and early lytic genes, but not late genes, suggesting roles for arginine and methionine metabolism in synthesis of lytic EBV genomes, the DNA templates for late gene expression. Interestingly, however, PALA induced expression of the EBV late genes p18/viral capsid antigen and gp350, even though we did not observe late gene expression with CRISPR CAD KO. PALA is a rationally synthesized analog of the transition-state intermediate produced by CAD conversion of carbamoyl phosphate and aspartic acid into carbamoyl aspartate (97). Although PALA is a potent CAD inhibitor with an estimated Km of ~2 × 10^−5^ M (98), it is nonetheless competitive with carbamoyl phosphate. We therefore hypothesize that partial CAD inhibition by PALA is sufficient to constrain *de novo* pyrimidine biosynthesis, as evidenced by uridine rescue, but still allows for some enzyme activity needed for lytic viral genome synthesis and late gene expression.

With regard to the mechanism by which inhibition of CAD or *de novo* pyrimidine biosynthesis triggers EBV reactivation, our data suggest that arginine restriction triggers DNA damage signaling. This may arise from imbalanced nucleotide pools and replication stress (99, 100) and/or from increased mitochondrial production of ROS, which can oxidize nucleotides, can directly activate ATM (101), and can cause single-strand DNA breaks (84). Amino acid restriction, including that of arginine, inhibits mTOR (102). Of these, our data favor nucleotide imbalance as the key factor, at least in EBV+ Burkitt cells, because arginine restriction activated both ATM and ATR pathways, and arginine restriction effects on lytic gene expression could be partially reversed by ATR and/or ATM inhibition, by uridine and/or cytidine supplementation, while ROS levels remain mostly unchanged. Uridine and cytidine can be metabolized to each of the other pyrimidine nucleotides. By contrast, CMP is not readily converted to UMP (103). Therefore, it is likely that arginine and CAD support of pyrimidine nucleotide pools is necessary for EBV latency, rather than support of UMP alone. Importantly, ATM- and ATR-driven p53 upregulation can reactivate EBV within latently infected gastric cancer cells (104). Notably, EBV+ Burkitt arginine restriction also highly altered glycolysis and pentose phosphate pathway metabolites. Since each of these pathways are highly connected to nucleotide biosynthesis, it is possible that arginine restriction effects on either or both of them also contributed to nucleotide pool imbalance.

How arginine restriction inhibits CAD and *de novo* pyrimidine biosynthesis warrants future investigation. Since addition of ornithine or citrulline at levels present for arginine in RPMI did not block arginine restriction-driven EBV lytic protein expression, we postulate that arginine regulates CAD through an mTOR-independent pathway. We speculate that arginine restriction may alter activity of the mitogen-activated protein kinases (MAPK), extracellular signal-regulated kinase 1 and 2 (ERK1/2). In support, MAPK can phosphorylate CAD threonine 456 to promote cell proliferation and *de novo* pyrimidine biosynthesis (105, 106). Interestingly, EBV oncoprotein LMP1 activates ERK-MAPK (107, 108) signaling. There is significant interest in EBV lytic induction therapies, and recent clinical trials have shown early but promising results (32, 109). Exploiting EBV-infected cell metabolic vulnerabilities provides an opportunity to sensitize EBV-infected cells to the antiviral ganciclovir, whose cytotoxicity is activated by the EBV early lytic kinase BGLF4 (26). Furthermore, dependence on extracellular arginine is a metabolic vulnerability in many types of cancers, and PEGylated arginase is in clinical development for multiple types of cancers (110). It will therefore be of interest to test the extent to which arginase counteracts growth of tumors in EBV+ Burkitt or gastric carcinoma xenograft models, either alone or in combination with hypomethylation and/or ganciclovir.

We recently found that restriction of extracellular methionine altered the EBV epigenome, reducing CpG DNA methylation and altering several histone epigenetic marks (76). While arginine restriction likewise induced EBV immediate-early and early lytic antigens, we instead found that arginine restriction increased global EBV+ Burkitt 5-methylcytosine levels, though interestingly had variable effects on EBV genomic DNA methylation. We speculate that this property underlies our observation that arginine restriction and DNA hypomethylation by UHRF1 KO or by decitabine together more highly induced EBV lytic reactivation than either did alone. Interestingly, however, as we previously described (76, 96), Burkitt cells in the latency I state appear to have a stochastic choice of switching to lytic reactivation versus to latency III. Yet, whereas decitabine or UHRF1 KO each trigger a Burkitt subpopulation to induce LMP1 and its cellular target ICAM-1, PALA did not induce ICAM-1 expression and instead preferentially induced gp350, indicative of lytic reactivation.

We note that plasma membrane gp350 staining likely underestimated the extent of lytic reactivation, given that cells with an abortive lytic cycle may have expressed immediate-early and early EBV lytic proteins but not late proteins. Since PALA and decitabine induced most cells to either induce the lytic cycle or LMP1, an intriguing approach would be to follow PALA/decitabine treatment with ganciclovir and to then adoptively transfer LMP1-specific cytotoxic T cells (CTL). This might serve to improve responses beyond those observed with decitabine and CTL therapy alone, where T cells homed to low dose decitabine-treated tumors and exerted a degree of anti-tumor effect at 3 weeks post-infusion (96).

In summary, EBV-infected tumor cells rely on arginine metabolism to support the maintenance of EBV latency in both B and epithelial cell contexts. Mechanistically, extracellular arginine supports CAD-driven *de novo* pyrimidine synthesis to supply sufficient UMP, which can then be converted to all of the pyrimidine nucleotides. Arginine restriction therefore caused nucleotide pool imbalance and DNA damage, which in turn triggered reactivation. Arginine restriction or CAD inhibition and DNA hypomethylation together induced higher levels of EBV reactivation, suggesting therapeutic approaches.

## MATERIALS AND METHODS

### Cell lines, culture, and growth curve analysis

293T cells were purchased from ATCC and cultured in Dulbecco modified Eagle medium (Gibco) with 10% FBS (Thermo Fisher). Burkitt lymphoma cell lines P3HR-1, EBV− Akata, EBV− MUTU, MUTU I, Rael, EB3, and EBV+ gastric cancer cell line SNU719 were cultured in RPMI 1640 (Gibco, Life Technologies) supplemented with 10% fetal bovine serum (Gibco). P3HR-1 that can be induced to lytic reactivation with 4-HT contains ZHT/RHT, which are BZLF1/BRLF1 fused to a modified estrogen receptor 4-HT-binding domain (gift from Eric Johannsen). P3HR-1, MUTU I, and Rael expressing *Streptococcus pyogenes* Cas9 genes were generated previously by lentiviral transduction, followed by blasticidin selection at 5 μg/mL (111). Cells were tested routinely to be mycoplasma-free with the MycoAlert kit (Lonza).

For the single amino acid restriction screen, P3HR-1 cells were washed with phosphate-buffered saline (PBS) three times and then resuspended to 0.3 million/mL in a panel of RPMI 1640 media lacking individual amino acids (Thermo Fisher). The panel of RPMI 1640 media was reconstituted from amino acid-free RPMI 1640 powder (Thermo Fisher), with each amino acid added from stock solutions described in Table S4, and then supplied with 10% dialyzed FBS. Amino acid solutions were prepared as concentrated stocks from powder (see Table S3), except for L-glutamine, which was purchased as a 100x solution (Gibco). Amino acid stock solution concentrations and solvent details are listed in Table S4. After dissolving powders in solvent, amino acid stock solutions were filter sterilized with 0.2 μm Uniflo Syringe Filters, PES (Cytiva Whatman). When reconstituting amino acid-free RPMI 1640 from powder, the pH was not adjusted by adding 1 N HCl, but by adding cystine stock solution, which was dissolved in 1N HCl. For cystine-free amino acid, an equal volume of 1 N HCl was added. For arginine restriction, cells were cultured in RPMI 1640 Medium for SILAC (Thermo Fisher) with supplementation of normal media concentration (0.22 mM) of L-lysine and 10% dialyzed FBS, as RPMI 1640 medium for SILAC does not contain L-arginine or L-lysine. Catalog numbers of chemicals are listed in Table S3. For UMP, CMP, GMP, AMP supplementation, 10 μg/μL stock solutions were made in H_2_O and diluted 1:200 to make a final concentration of 50 μg/mL, then sterilized with Uniflo Syringe Filters, 0.2 μm PES (Cytiva Whatman). For cell growth curve, 10 mL culture in T25 flasks was mixed by pipetting, from which 1 mL culture was taken, centrifuged at 1,500 rpm for 5 min, and resuspended in 1 mL PBS. Cells were stained with 0.4% trypan blue (Thermo Fisher), and live cell numbers were counted by light microscopy.

### Immunoblot analysis

WCL samples were separated by SDS-PAGE, transferred onto nitrocellulose filters (Bio-Rad), blocked with TBST (Tris-buffered saline with Tween 20) containing 5% milk or 3% BSA (NEB), and then probed with primary antibodies at 4°C overnight, followed by secondary antibody (Cell Signaling Technology, catalog no. 7074, catalog no. 7076, catalog no. 7077; LI-COR Inc., catalog no. 926-32210, catalog no. 926-32219) incubation for 1 h at room temperature. Primary antibody dilutions are: anti-β-actin rat monoclonal antibody 1:4,000, anti-EBNA2 mouse antibody clone R3 1:2,000, anti-EBV Ea-D mouse monoclonal antibody (0261) 1:500, anti-BZLF1 mouse monoclonal antibody 1:500, Epstein-Barr virus p18 polyclonal antibody 1:5,000, anti-5-methylcytosine mouse monoclonal antibody 1:5,000, anti-ASS1 rabbit antibody 1:5,000, anti-CAD rabbit polyclonal antibody 1:2,500, anti-DHODH rabbit polyclonal antibody 1:2,000, anti-UHRF1 rabbit polyclonal antibody 1:2,000, anti-phospho-ATM (Ser1981) (D25E5) rabbit mAb 1:1,000, anti-phospho-Chk1 (Ser345) (133D3) rabbit mAb 1:1,000, anti-phospho-Chk2 (Thr68) (C13C1) rabbit mAb 1:1,000. Horseradish peroxidase (HRP)-conjugated secondary antibodies were diluted 1:4,000. For all HRP-conjugated secondary antibodies, ECL chemiluminescence (Millipore, catalog no. WBLUF0500) was used to develop HRP signals. All images were captured with a Li-Cor FC platform. All antibodies used in this study are listed in Table S3.

For DNA dot blot, DNA harvested by DNeasy Blood & Tissue Kit (Qiagen) from cells was hybridized on nitrocellulose membranes and blocked with TBST containing 5% milk. Membrane was then washed and blotted with anti-5 methyl-cytosine monoclonal antibody overnight or stained with 1 mg/mL ethidium bromide in PBS for 10 min. After further washing with TBST (for immunoblot) or PBS (for ethidium bromide staining) 5 min × 3, membranes were imaged as described above. Western blot densitometry was performed with Image Studio v.5.5. Equal area was selected for each band of the same protein, and relative protein abundances were standardized to corresponding β-actin load control (BioLegend, 664802) bands for SDS-PAGE blot or to ethidium bromide for dot blot.

### Quantification of EBV genome copy number by PCR analysis

Total DNA from 0.5 to 1 × 10^6^ cells was extracted with DNeasy Blood & Tissue Kit (Qiagen). Then, DNA extracted from each sample was diluted to 10 ng/mL and was then subjected to qPCR analysis with primer pairs targeting the EBV BALF5 gene and the host *ACTB* (β-actin) gene (Table S3). Quantitative real-time PCR was performed using Power SYBR Green PCR Master Mix (Applied Biosystems) on a CFX96 Touch Real-Time PCR Detection System (Bio-Rad). Viral DNA copy number was calculated by standardizing to *ACTB* or interpolation of Cq values to a standard curve generated by serial dilutions of the pHAGE-BALF5 cDNA plasmid (25 ng/mL).

### Immunofluorescent confocal imaging

Briefly, 5 × 10^5^ cells suspended in PBS were allowed to dry on glass slides and then fixed with 4% paraformaldehyde in PBS for 10 min. Cells were then washed and permeabilized with 0.1% Triton X in PBS for 5 min. Cells were then blocked with PBS containing 1% low-IgG BSA (MP Bio) overnight at 4°C. Cells were incubated with primary antibodies for 1 h at 37°C, washed three times, and incubated in secondary fluorescent antibody for 1 h at 37°C before being washed three times again and mounted with ProLong Gold AntiFade Mountant with DAPI (Thermo Fisher). Primary antibody dilutions are: anti-EBV Ea-D (BMRF1) mouse monoclonal antibody (0261) 1:200, anti-phospho-histone H2A.X (Ser139) antibody, clone JBW301, 1:200. Secondary antibodies (Invitrogen) were diluted 1:1,000. Confocal images were taken on a Zeiss LSM 800 instrument. BMRF1+ cells were counted by eye with the assistance of FIJI ImageJ software.

### Flow cytometry analysis

All cells were washed once with PBS before staining. For 7-AAD and annexin V flow cytometry analysis, 1 × 10^6^ cells were stained in 100 μL of staining buffer that contained FITC-conjugated anti-annexin V (1:20) and 5 μg/mL 7-AAD for 15 min. Annexin V and 7-AAD were diluted in annexin V binding buffer, which contains 10 mM HEPES pH 7.4, 140 mM NaCl, and 2.5 mM CaCl_2_. Before flow cytometry analysis, the total volume was expanded to 500 μL with annexin V binding buffer. For gp350 and ICAM1 flow cytometry analysis, cells were incubated with Cy5-conjugated anti-gp350 antibody (1:800) and PE-conjugated anti-CD54 antibody (1:800) in PBS containing 2% FBS for 45 min at 4°C. Cells were then washed twice with PBS containing 2% FBS and resuspended in 500 μL PBS containing 2% FBS before flow cytometry analysis. For Zombie Aqua and BMRF1 flow cytometry analysis, 1 × 10^6^ cells were stained in 100 μL PBS with Zombie Aqua (1:2,000) for 20 min. Cells were then fixed and permeabilized in Intracellular Fixation & Permeabilization Buffer Set (eBioscience) for 1 h. Fixed cells were then stained in 100 μL perm/wash buffer (BD Biosciences) containing BMRF1 antibody (Santa Cruz; 1:200) for 45 min, followed by washing. Cells were then stained with anti-mouse Alexa-647 secondary antibody (1:1,000) in PBS for 45 min at room temperature. For ROS measurement, at the time of measurement, cells were seeded at 3 × 10^5^/mL in RPMI 1640 media with 4 μM H_2_DCFDA (Life Technologies) for 30 min before washing with PBS twice and analysis. For mitochondrial ROS measurement, at the time of measurement, cells were seeded at 3 × 10^5^/mL in PBS with 500 nM MitoSOX (Thermo Fisher) for 30 min, washed twice with PBS, and analyzed by flow cytometry, which was performed on a BD FACSCalibur instrument. FACS data were analyzed with FlowJo V10 software.

### RNA-seq analysis

Frozen P3HR-1 cell pellets with 2 × 10^6^ cells were submitted to Quintara Biosciences for RNA extraction, reverse transcription, library creation, and sequencing. Adaptor-trimmed Illumina reads for each individual library were mapped back to the human GRCh38.p14 transcriptome assembly or to the EBV Akata genome (GCA_900004315.1), using STAR v.2.7.10a (112). Feature Counts were used to estimate the number of reads mapped to each contig. Only transcripts with at least 10 counts combined in all samples were used. Raw FASTQ and read count files were submitted to NIH Gene Expression Omnibus (GSE324976). DESeq2 v.1.48.2 was used to evaluate differential gene expression (DE) (113). DESeq2 uses a negative binomial distribution to account for overdispersion in transcriptome data sets. It uses a conservative analysis that relies on a heuristic approach. Each DE analysis used pairwise comparison between the experimental and control groups. Log2 transformation was used for counts per million. *P*-value and log_2_ fold-change values were generated with DESeq2 under default settings with Benjamini-Hochberg correction. Differentially expressed genes were identified using *P*-value <0.05 and absolute fold change >2 cutoffs. Volcano plots were built with GraphPad Prism 11. Differentially expressed genes were subjected to KEGG 2011 (human) pathway analysis (114). The Top 10 KEGG pathways were visualized using GraphPad Prism 11.

### Metabolomic analysis

For profiling the metabolome of arginine-restricted EB3 cells, 1 × 10^7^ cells were seeded into each T75 flask (five flasks for each condition) with 20 mL RPMI 1640 Medium for SILAC, supplied with 10% dialyzed FBS and 0.22 mM L-lysine. L-arginine was added to 1.15 mM for the control (100% Arg) group. Three days after seeding, media were refreshed for all groups by pelleting cells at 1,500 rpm for 10 min and resuspension in an equal volume of fresh respective media. Cells in the control group were split 1:5 to account for cell proliferation. Five days after seeding, 3 × 10^6^ total cells from each T75 flask were seeded into a T25 in 10 mL respective media 2 h prior to intracellular metabolite extraction. For profiling the metabolome of arginine-restricted P3HR-1 cells, cells were treated the same as EB3 cells, except with three replicates. For profiling the metabolome of CAD-knockout P3HR1, 3 × 10^6^ control or CAD-depleted cells were collected 9 days after CRISPR editing. Cells were seeded in 10 mL fresh RPMI 1640 media supplied with 10% FBS 2 h prior to intracellular metabolite extraction.

For intracellular metabolite extraction, cells were washed with 5 mL of room temperature PBS. Then, pellets were resuspended in 1 mL of dry ice-cold 80% methanol, incubated at −80°C for 30 min, and centrifuged at 21,000 × *g* for 5 min at 4°C. Supernatants were collected in pre-chilled tubes and stored at −80°C. For media metabolite extraction, replete or arginine-free RPMI 1640 SILAC supernatant from P3HR-1 cell cultures was centrifuged at 1,200 × *g* to remove cells, then filtered with Uniflo Syringe Filters, 0.2 μm PES (Cytiva Whatman) to remove debris. Then, 50 μL was taken from each replicate and mixed with 200 μL LC-MS grade methanol and incubated at −80°C for 30 min and centrifuged at 21,000 × *g* for 5 min at 4°C. Three replicates for P3HR-1 cells or P3HR-1 cell culture media or six EB3 cell replicates were used for each treatment. On the day of analysis, supernatants were incubated on ice for 20 min, clarified by centrifugation at 21,000 × *g* at 4°C, and dried down with a speed vacuum concentrator (Savant SPD 1010, Thermo Fisher Scientific). Samples were re-suspended in 100 μL of 60/40 acetonitrile/water, vortexed, sonicated in ice-cold water, and incubated on ice for 20 min. Following centrifugation at 21,000 × g for 20 min at 4°C, supernatants were collected for pooled QC. Metabolite profiling was performed at the Beth Israel Deaconess Mass Spectrometry Core. Samples were re-suspended using 20 µL HPLC-grade water for mass spectrometry. Five to seven microliters were injected and analyzed using a hybrid 6500 QTRAP triple quadrupole mass spectrometer (AB/SCIEX) coupled to a Prominence UFLC HPLC system (Shimadzu) via selected reaction monitoring (SRM) of a total of 300 endogenous water-soluble metabolites for steady-state analyses of samples. Some metabolites were targeted in both positive and negative ion mode for a total of 311 SRM transitions using positive/negative ion polarity switching. ESI voltage was +4,950 V in positive ion mode and –4,500 V in negative ion mode. The dwell time was 3 ms per SRM transition, and the total cycle time was 1.55 s. Approximately 9–12 data points were acquired per detected metabolite. Samples were delivered to the mass spectrometer via hydrophilic interaction chromatography using a 4.6 mm i.d. × 10 cm Amide XBridge column (Waters) at 400 μL/min. Gradients were run starting from 85% buffer B (HPLC-grade acetonitrile) to 42% B from 0 to 5 min; 42% B to 0% B from 5 to 16 min; 0% B was held from 16 to 24 min; 0% B to 85% B from 24 to 25 min; 85% B was held for 7 min to re-equilibrate the column. Buffer A was comprised of 20 mM ammonium hydroxide/20 mM ammonium acetate (pH = 9.0) in 95:5 water:acetonitrile. Peak areas from the total ion current for each metabolite SRM transition were integrated using MultiQuant v.3.0.2 software (AB/SCIEX). Metabolites with CV <30% in pooled QC were used for the statistical analysis. The quality of integration for each metabolite peak was reviewed. Metabolites with *P*-values <0.05 and log_2_ fold change >1 or <−1 were used for pathway analysis using MetaboAnalyst 5.0 (https://www.metaboanalyst.ca/MetaboAnalyst/ModuleView.xhtml). Heatmaps of metabolites in the pathways were generated by feeding *Z*-score values into Morpheus software (https://software.broadinstitute.org/morpheus/).

A separate cell pellet from each intracellular metabolite extraction sample was then quantified for protein content using a BCA assay kit (Thermo Fisher Scientific). Protein content was calculated by interpolation of OD_562_ values to a standard curve generated by serial dilutions of bovine serum albumin provided in the kit. Protein content ratios between samples were calculated by normalization, which was used to guide the standardization of metabolites peaks. Metabolites with *P*-values <0.05 and log_2_ fold change >1 or <−1 were used for pathway analysis using MetaboAnalyst 6.0.

### CRISPR-Cas9 mutagenesis

CRISPR-Cas9 editing was performed as previously described (111). Briefly, Broad Institute pXPR-011 GFP-targeting, sgRNA non-targeting control (BRDN0004438510), Avana or Brunello library sgRNAs were cloned into shuttle vector lentiGuide-Puro (Addgene, #52963) or pRDA_355 (Addgene, #187159). Production of lentivirus and transduction of target cells for CRISPR-Cas9 gene editing were described before (115). Shuttle vector (500 ng), packaging vectors psPAX2 (400 ng), and VSV-G (150 ng) were transfected into 0.8 million 293T cells in a well of a six-well plate (Corning) with TransIT-LT1 Transfection Reagent (Mirus) according to the manufacturer’s protocol. Twelve hours post-transfection, cell culture media was changed to RPMI-1640 with 10% FBS. 293T supernatants were harvested 48 and 72 h post-transfection and were added onto target cells that were seeded at 3 × 10^5^ cells/mL. Cells were selected by puromycin (3 mg/mL), which was added 48 h post-transduction. For CRISPR/Cas9 knockout using pRDA_355, doxycycline (Sigma) was added to 200 ng/mL to induce the expression of sgRNA. Cell pellets were harvested 48 h post-CRISPR editing and analyzed with immunoblot as described above to confirm the loss of protein expression.

### EBV genome FISH

Cells were washed with PBS and incubated 15 min at RT in 500 μL of buffer containing 0.2 N (0.05 M) acetic acid and 0.02 N (0.02 M) HCl/Tris HCl 0.1 M pH 3.7. Cells were then dried on slides at 37°C for 2 h, followed by 4% paraformaldehyde fixation and 0.5% Triton X-100 permeabilization (10 min each). For staining, cells were incubated in 2× saline-sodium citrate (SSC; Invitrogen) with 100 μg/mL RNase A at 37°C for 2 h and washed with 0.1 M Tris-HCl (pH 7.5, wash once), 0.1 M Tris-HCl (pH 7.5)/0.1 M glycine (incubate 5 min), and 2× SSC (wash twice). EBV probe (Enzo), which contains mixed probes that target the 3 kb Bam HI “W” region, that is, the internal repeat, was diluted to 1 μg/mL in FISH buffer (Enzo) and denatured at 95°C for 10 min, followed by incubation on cells on top of an 85°C heat block for 6 min, then at 37°C overnight. Cells were incubated in 0.1× SSC/0.1% Tween/1% BSA buffer on top of a 42°C heat block for 15 min, which was repeated twice, followed by wash with 0.1× SSC (once) and Tris-HCl (0.1 M, pH 7.5) (three times). Then streptavidin-conjugated Alexa 647 (1:1,000) was diluted in 1× PBS/0.1% Tween at 37°C for 1 h to allow conjugation. Cells were then washed and mounted with ProLong Gold Antifade Mountant with DAPI. Confocal images were taken on a Zeiss LSM 800 instrument. In latent cells, episome puncta were quantified using the local maxima package in FIJI ImageJ software, using DAPI-defined ROI as nuclei.

### Comet assay

Comet assays were conducted as described (85). Briefly, glass slides were pre-coated with 1% ultra-low gelling temperature agarose in distilled water and dried for >1 h to allow for better adhesion. P3HR-1 cells were resuspended at 1 × 10^5^/mL in PBS, 0.4 mL of which was then mixed with 1.2 mL 1% ultra-low gelling temperature agarose in distilled water (pre-heated to melt and stored in a 40°C water bath). Cells were allowed to adsorb onto the pre-coated glass slides. After agar solidification, slides were incubated in A1 buffer (1.2 M NaCl, 100 mM EDTA, 0.1% sodium lauryl sarcosinate, 0.26 M NaOH) overnight, and incubated in A2 buffer (0.03 M NaOH, 2 mM EDTA) for 20 min. Slides were then subjected to electrophoresis in A2 buffer at 21 V for 25 min. Slides were then neutralized in water and stained with 2.5 μg/mL propidium iodide (Thermo Fisher) for 20 min, followed by another wash with water. DNA fluorescence images were captured on a Zeiss LSM 800 instrument. Comet tail moments were calculated using the OpenComet package in FIJI ImageJ software.

### MeDIP and qPCR

Genomic DNA was purified using the Blood and Cell Culture DNA Mini Kit (Qiagen) and then used for MeDIP analysis, using the MagMeDIP kit (Diagenode catalog no. C02010021), according to the manufacturer’s protocol. qPCR assays were then performed as described above.

### Software/data presentation

Graphs were made using GraphPad Prism 11. Schematic models were made using BioRender.

## SUPPLEMENTAL MATERIAL

Supplemental figures mbio.00933-26-s0001.pdf Figures S1 to S7.

Table S1 mbio.00933-26-s0002.xlsx LC-MS/MS raw data of EB3 cells.

Table S2 mbio.00933-26-s0003.xlsx LC-MS/MS raw data of P3HR-1 cell cultures.

Table S3 mbio.00933-26-s0004.docx Reagents, cell lines, sgRNAs, and qPCR primers used in this study.

Table S4 mbio.00933-26-s0005.docx Amino acid stock solutions used in amino acid supplementation.

Table S5 mbio.00933-26-s0006.xlsx Metabolite LC-MS peak heights of P3HR1 culture media.

ASM does not own the copyrights to Supplemental Material that may be linked to, or accessed through, an article. The authors have granted ASM a non-exclusive, world-wide license to publish the Supplemental Material files. Please contact the corresponding author directly for reuse.
