# Supplementary material for: Arginine metabolism supports de novo pyrimidine biosynthesis to block DNA damage and maintain Epstein-Barr virus latency
Source: mBio. 2026 Jun 15;17(7):e00933-26. doi: 10.1128/mbio.00933-26 (PMC13343950; doi:10.1128/mbio.00933-26)
Supplement: Supplemental figures — Figures S1 to S7. [file mbio.00933-26-s0001.pdf]

Figure S1

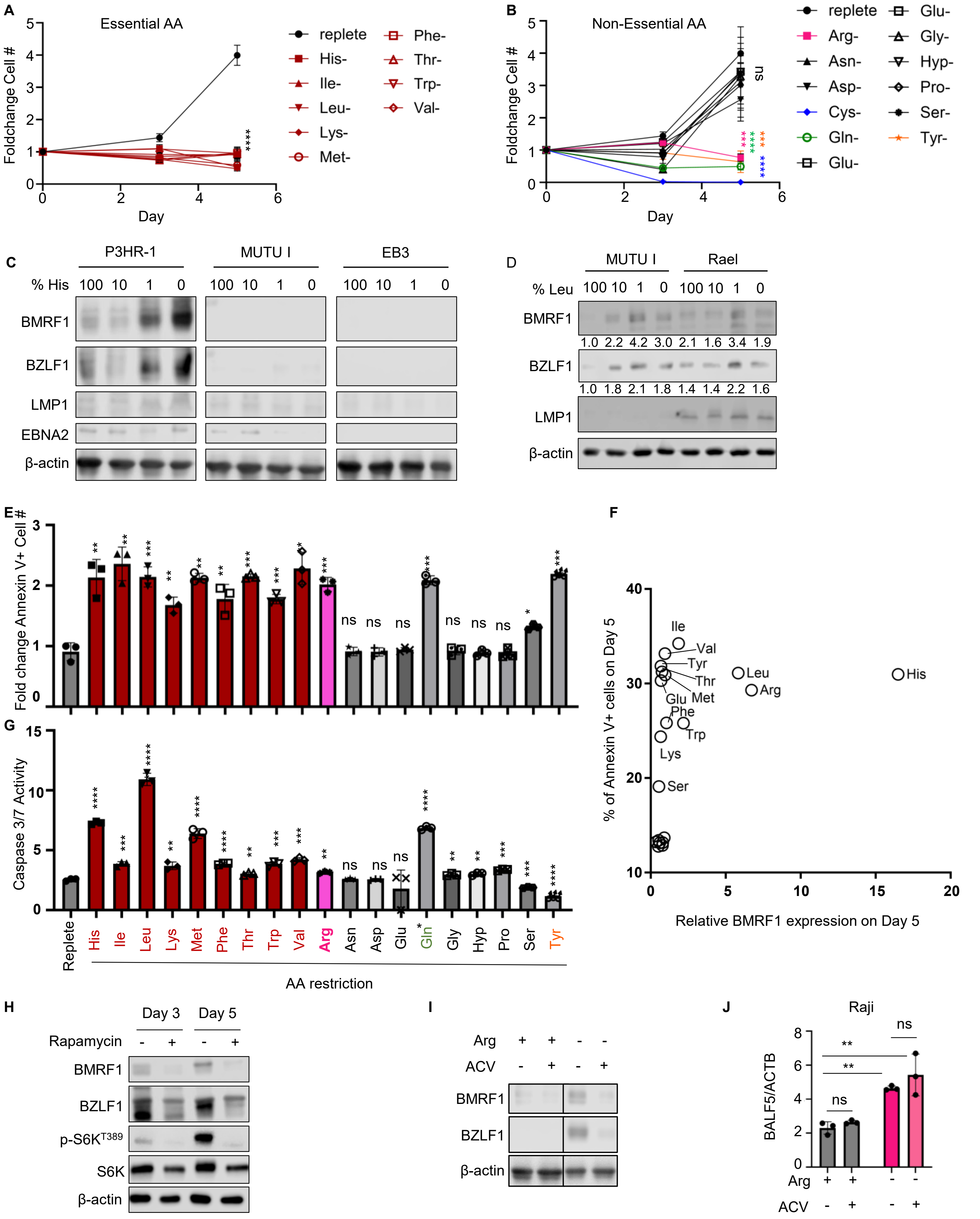
A
Essential AA
Foldchange Cell #
Day
replete
His-
Ile-
Leu-
Lys-
Met-
Phe-
Thr-
Trp-
Val-
****
B
Non-Essential AA
Foldchange Cell #
Day
ns
replete
Arg-
Asn-
Asp-
Cys-
Gln-
Glu-
Glu-
Gly-
Hyp-
Pro-
Ser-
Tyr-
C
P3HR-1
MUTU I
EB3
% His
100 10 1 0
BMRF1
BZLF1
LMP1
EBNA2
β-actin
D
MUTU I
Rael
% Leu
100 10 1 0
BMRF1
1.0 2.2 4.2 3.0 2.1 1.6 3.4 1.9
BZLF1
1.0 1.8 2.1 1.8 1.4 1.4 2.2 1.6
LMP1
β-actin
E
Fold change Annexin V+ Cell #
ns
F
% of Annexin V+ cells on Day 5
Relative BMRF1 expression on Day 5
Ile
Val
Tyr
Thr
Glu
Met
Phe
Trp
Lys
Ser
Leu
Arg
His
G
Caspase 3/7 Activity
Replete
His
Ile
Leu
Lys
Met
Phe
Thr
Trp
Val
Arg
Asn
Asp
Glu
Gln
Gly
Hyp
Pro
Ser
Tyr
AA restriction
H
Day 3
Day 5
Rapamycin
BMRF1
BZLF1
p-S6K T389
S6K
β-actin
I
Arg
ACV
BMRF1
BZLF1
β-actin
J
Raji
BALF5/ACTB
ns
Arg
ACV

Figure S1. Arginine or histidine restriction effects on EBV+ Burkitt cells, related to Figure 1.

(A) Effects of essential amino acid (AA) restriction on P3RH-1 proliferation. Shown are mean ± standard error of the mean (SEM) fold-change in live cell number of cells grown in replete media versus in media lacking the indicated essential amino acid, normalized to cell numbers at day 1. (B) Effects of non-essential AA restriction on P3RH-1 proliferation. Although considered non-essential amino acids that do not have be acquired by diet, restriction of either cystine, arginine, glutamine or tyrosine significantly impaired P3HR-1 proliferation. (C) Histidine restriction effects on EBV lytic reactivation. Immunoblot analysis of WCL from P3HR-1, MUTU I or EB3 Burkitt cells cultured in media for 5 days with the indicated histidine level, where 100% histidine refers to the typical RPMI histidine concentration (97μM). (D) Leucine restriction effects on EBV lytic reactivation. Immunoblot analysis of WCL from MUTU I or Rael Burkitt cells cultured in media for 5 days with indicated leucine levels, where 100% leucine refers to the typical RPMI leucine concentration (38μM). (E) Analysis of amino acid restriction effects on cell death. Shown are the relative mean ± SD fold-change of % Annexin V+ values from n = 3 replicates of FACS analysis from P3HR-1 cells cultured in the indicated replete versus media restricted for the indicated amino acid for 5 days. The value of cells cultured in replete media was set to one. (F) Analysis of the correlation between annexin V positivity and BMRF1 expression. Shown are the mean BMRF1 band intensity (x-axis) from Figure 1C versus the mean %Annexin V+ cells from Figure S1C (y-axis). (G) Analysis of amino acid restriction effects on apoptosis. Shown are the mean ± SD values from n=3 replicates of caspase 3/7 activity from cells grown as in (C). Values were normalized by cell number. (H) Immunoblot analysis of immediate early BZLF1, early BMRF1, phospho-p70 S6 kinase (Thr389), total p70S6 kinase or load control β-actin using WCL from P3HR-1 cells cultured in media with vehicle or 10nM rapamycin for 3 or 5 days. (I) Immunoblot analysis of WCL from P3HR-1 cells cultured in replete or arginine-free media for 5 days, with or without 100 μg/ml acyclovir (ACV) to block EBV lytic DNA replication. (J) Mean ± SD EBV intracellular genome copy number from qPCR analysis of n=3 biological replicates of Raji Burkitt cells grown in replete or arginine-free media for 5 days, with or without 100 μg/ml ACV. Raji cells have an EBV genomic deletion that prevents lytic DNA synthesis. Student's t-test was performed for (A-B), (E) and (G), two-way ANOVA was performed for (J), with ****p<0.0001, ***p<0.001, **p< 0.01, *p<0.05.

Figure S2

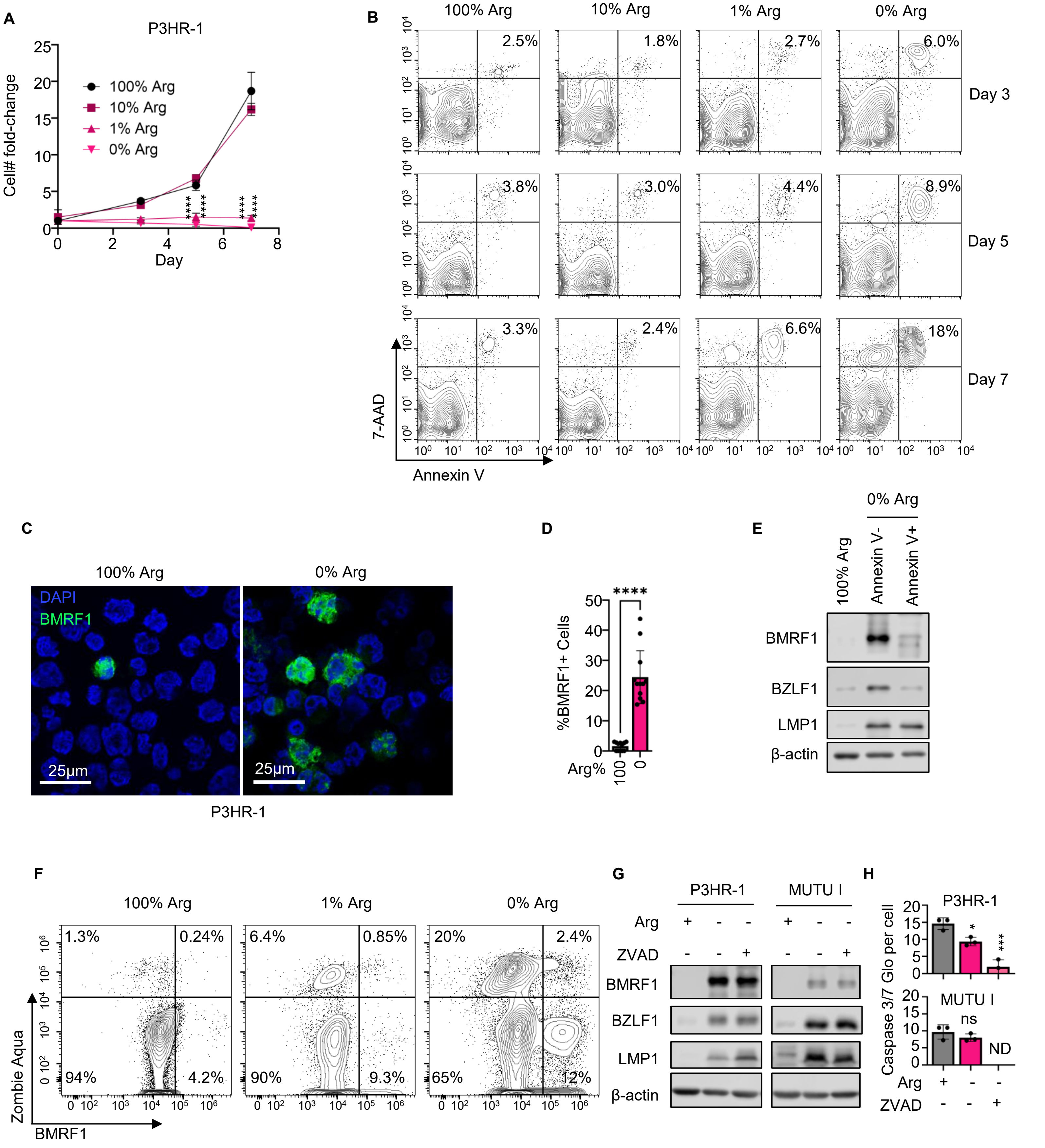

A
P3HR-1
Cell# fold-change
100% Arg
10% Arg
1% Arg
0% Arg
Day
B
100% Arg
10% Arg
1% Arg
0% Arg
Day 3
Day 5
Day 7
7-AAD
Annexin V
C
100% Arg
0% Arg
DAPI
BMRF1
25µm
P3HR-1
D
%BMRF1+ Cells
Arg%
E
0% Arg
100% Arg
Annexin V-
Annexin V+
BMRF1
BZLF1
LMP1
β-actin
F
100% Arg
1% Arg
0% Arg
Zombie Aqua
BMRF1
G
P3HR-1
MUTU I
Arg
ZVAD
BMRF1
BZLF1
LMP1
β-actin
H
P3HR-1
MUTU I
Caspase 3/7 Glo per cell
ns
ND
Arg
ZVAD

Figure S2. Analysis of arginine restriction effects on P3HR-1 Burkitt growth and survival.

(A) Analysis of arginine restriction effects on P3HR-1 Burkitt B cell proliferation. Growth curves of P3HR-1 cells cultured in media with the indicated arginine (Arg) concentration. 100% arginine refers to the RPMI arginine concentration of 115 μM. Mean ± SD values from n = 4 replicates are shown. (B) FACS analysis of cells in (A). (C) Confocal immunofluorescence analysis of BMRF1 expression in P3HR-1 cells cultured in replete or arginine-free media for 5 days. (D) Mean ± SD percentages of BMRF1+ cells from n=13 fields as in (C). (E) Analysis of the correlation between cell death and EBV lytic gene expression. Immunoblot analysis of WCL from P3HR-1 cells cultured in arginine-free media for 5 days and then FACSorted for Annexin V positivity vs negativity. WCL from unsorted P3HR-1 cultured in replete media was used as control. (F) Analysis of the correlation between cell death and EBV lytic gene expression. Shown is FACS analyses of P3HR-1 cells cultured in media with the indicated % of RPMI arginine levels for 5 days and then stained with Zombie Aqua and for intracellular BMRF1, representative of n=3 replicates. (G) Immunoblot analysis of WCL from P3HR-1 cells cultured in replete or arginine-free media in DMSO vehicle or pan-caspase inhibitor 20μM Z-VAD-FMK for 5 days. (H) Mean ± SD caspase 3/7 activity from n=3 biological replicates of cells cultured as in (G). One-way ANOVA was performed for each comparison in (A) and (H); Student's T-test was performed in (D). **** $p<0.0001$, *** $p<0.001$, ** $p<0.01$, * $p<0.05$.

Figure S3

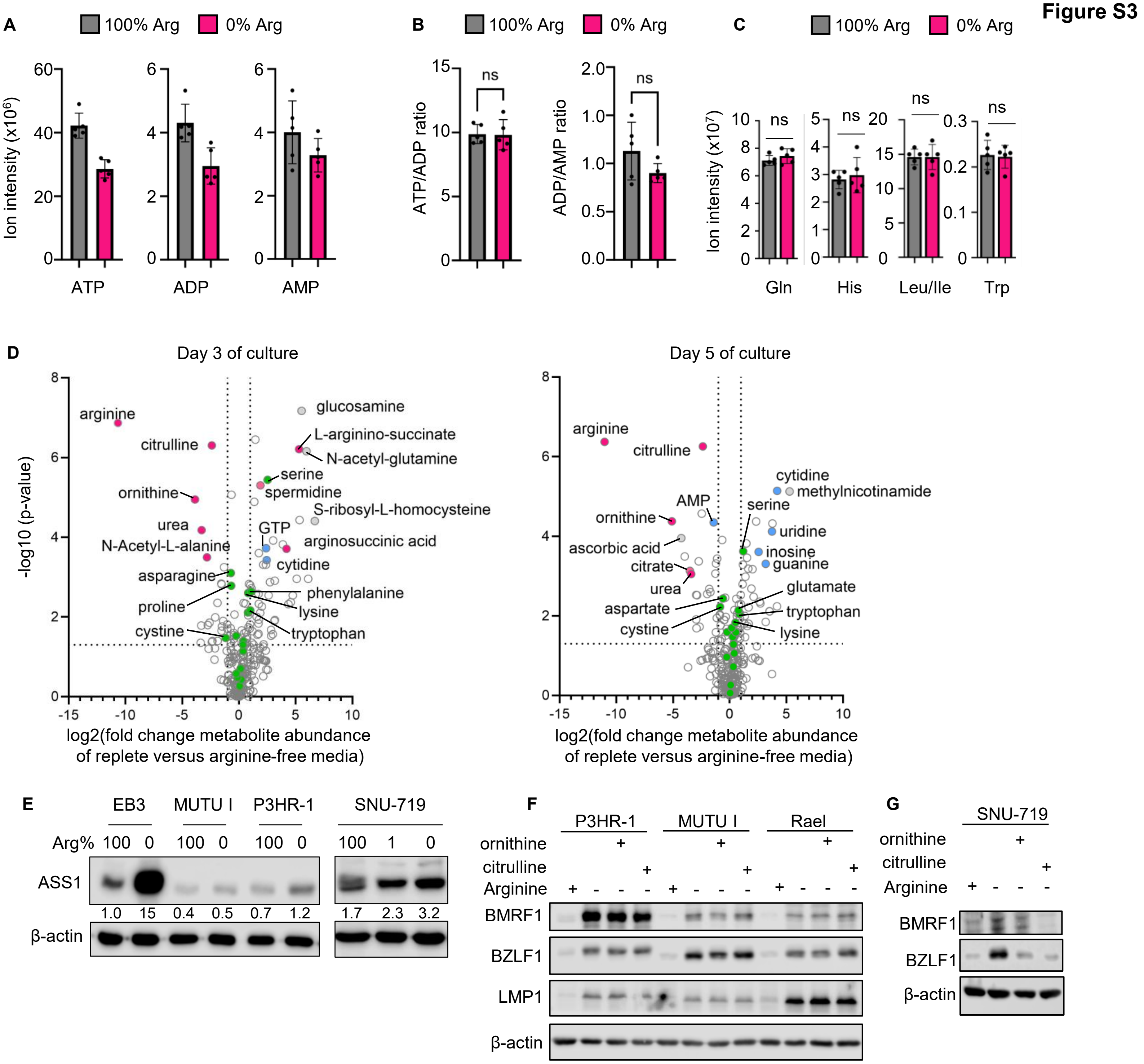

A
100% Arg
0% Arg
Ion intensity (x10^6)
ATP
ADP
AMP
B
ATP/ADP ratio
ADP/AMP ratio
ns
C
Ion intensity (x10^7)
Gln
His
Leu/Ile
Trp
D
Day 3 of culture
Day 5 of culture
-log10 (p-value)
log2(fold change metabolite abundance of replete versus arginine-free media)
arginine
citrulline
ornithine
urea
N-Acetyl-L-alanine
asparagine
proline
cystine
glucosamine
L-arginino-succinate
N-acetyl-glutamine
serine
spermidine
S-ribosyl-L-homocysteine
GTP
arginosuccinic acid
cytidine
phenylalanine
lysine
tryptophan
AMP
ascorbic acid
citrate
aspartate
methylnicotinamide
uridine
inosine
guanine
glutamate
E
EB3
MUTU I
P3HR-1
SNU-719
Arg%
ASS1
β-actin
1.0 15 0.4 0.5 0.7 1.2
1.7 2.3 3.2
F
Rael
ornithine
citrulline
Arginine
BMRF1
BZLF1
LMP1
G

Figure S3. Arginine restriction effects on ATP, ADP and AMP, related to Figure 3.

(A) Analysis of arginine restriction effects on adenosine nucleotide levels, from LC/MS metabolomic analysis of EB3 cells grown in replete (100% Arg) vs arginine free (0% Arg) media, as in Figure 3A. Shown are mean ± SD values from n=5 replicates. (B) Analysis of arginine restriction effects on the ATP/ADP or ADP/AMP ratios, from the LC/MS analysis presented in Figure 3A. Shown are mean ± SD values from n=5 replicates. (C) Analysis of arginine restriction effects on extracellular amino acid levels. Shown are mean ± SD values from n=5 LC/MS metabolomic analysis of EB3 cells grown in replete (100% Arg) vs arginine free (0% Arg) media, as in Figure 3A, where media was refreshed on day 3. (D) Analysis of arginine restriction effects on metabolites in Burkitt cell culture media. Shown are volcano plots of LC/MS metabolomic analysis of EBV+ P3HR-1 cells cultured arginine free vs arginine-replete media for 5 days, from n=3 replicates. Higher fold change values indicate higher levels in media from cells grown under arginine free conditions. Arginine cycle metabolites are highlighted in pink; pyrimidine and purine related metabolites are highlighted in blue. Amino acids are highlighted in green. (E) Analysis of arginine restriction effects on ASS1 expression. Immunoblot analysis of WCL from the indicated EBV+ Burkitt or SNU-719 cells cultured in replete vs arginine free RPMI for 5 days. β-actin-normalized ASS1 signals are shown. (F, G) Analysis of arginine metabolite effects on EBV reactivation. Immunoblot analysis of WCL from Burkitt cells (F) and SNU-719 (G) cultured in replete or arginine-free media supplemented with PBS vehicle, or with arginine metabolites ornithine or citrulline (1.15mM) for 5 days.

Figure S4

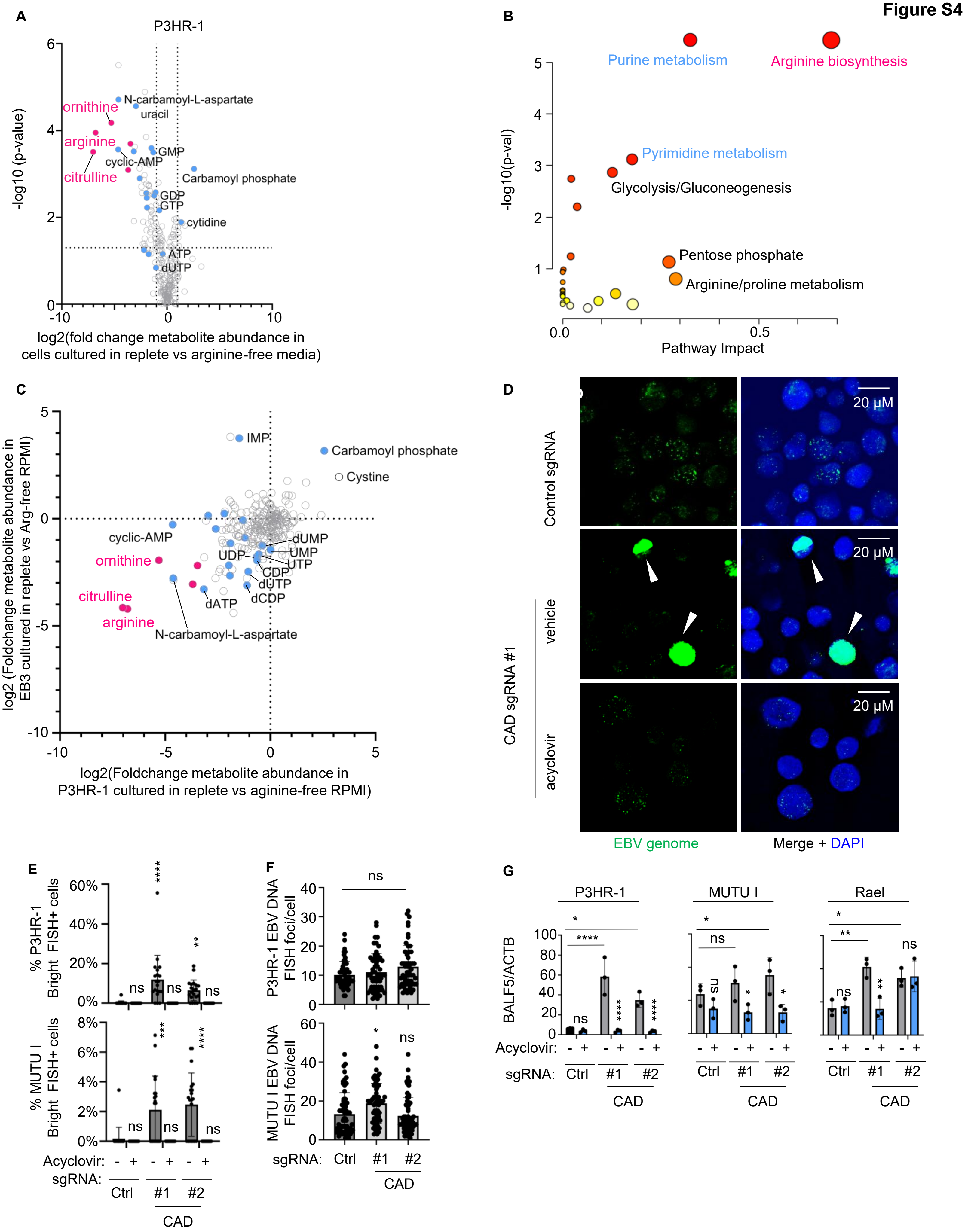
A
P3HR-1
-log10 (p-value)
log2(fold change metabolite abundance in cells cultured in replete vs arginine-free media)
ornithine
arginine
citrulline
N-carbamoyl-L-aspartate
uracil
GMP
cyclic-AMP
Carbamoyl phosphate
GDP
GTP
cytidine
ATP
dUTP
B
-log10(p-val)
Pathway Impact
Purine metabolism
Arginine biosynthesis
Pyrimidine metabolism
Glycolysis/Gluconeogenesis
Pentose phosphate
Arginine/proline metabolism
C
log2 (Foldchange metabolite abundance in EB3 cultured in replete vs Arg-free RPMI)
log2(Foldchange metabolite abundance in P3HR-1 cultured in replete vs aginine-free RPMI)
IMP
Carbamoyl phosphate
Cystine
cyclic-AMP
ornithine
citrulline
arginine
dUMP
UDP
UMP
UTP
CDP
dUTP
dATP
dCDP
N-carbamoyl-L-aspartate
D
Control sgRNA
CAD sgRNA #1
vehicle
acyclovir
20 μM
EBV genome
Merge + DAPI
E
% P3HR-1 Bright FISH+ cells
% MUTU I Bright FISH+ cells
Acyclovir:
sgRNA:
Ctrl
#1
#2
CAD
ns
F
P3HR-1 EBV DNA FISH foci/cell
MUTU I EBV DNA FISH foci/cell
sgRNA:
Ctrl
#1
#2
CAD
ns
G
P3HR-1
MUTU I
Rael
BALF5/ACTB
Acyclovir:
sgRNA:
Ctrl
#1
#2
CAD
ns

Figure S4. ***De novo*** **pyrimidine biosynthesis maintains EBV latency, related to Figure 4.**

(A) Metabolomic analysis of EBV+ P3HR-1 cells cultured in arginine-free vs replete RPMI for 5 days. Shown are foldchanges of metabolite abundance and p-values, calculated from n=3 replicates. Arginine related metabolites are highlighted in pink; pyrimidine and purine related metabolites are highlighted in blue. Higher foldchange indicate higher metabolite abundance in cells grown in arginine replete than arginine free media. (B) KEGG metabolic pathway analysis of metabolomic data as in (A). Metabolites were selected using a FDR<0.05 cutoff and pathway impact values were computed by MetaboAnalyst 3.0 topological analysis. (C) Volcano plot visualization of log2 foldchange in metabolite abundance from LC/MS analysis of EBV+ P3HR-1 cells (x-axis) versus of EBV+ EB3 cells (y-axis) cultured in arginine replete vs free media for 5 days. Arginine cycle metabolites are highlighted in pink; pyrimidine and purine related metabolites are highlighted in blue. (D) EBV genomic DNA fluorescence in situ hybridization (FISH) analysis of Cas9+ P3HR-1 that expressed control or *CAD* targeting sgRNA, ± 100 μg/ml ACV. Arrow heads indicate cells with robust EBV FISH signals. (E) Mean ± SD percentages of Cas9+ P3HR-1 (top) or MUTU I (bottom) cells with bright EBV DNA FISH signals. Cas9+ P3HR1 or MUTU I expressed control or *CAD* targeting sgRNA for 6 days and were treated with 100 μg/ml ACV as indicated. Data are from 20 randomly selected panels. (F) Mean ± SD number of EBV genome DNA foci in cells without bright FISH nuclear foci. 60 randomly chosen cells were quantified using local maxima package in ImageJ. (G) Analysis of CAD KO effects on EBV genome copy number. Mean ± SD *ACTB* normalized intracellular EBV genome copy number of cells as in (E). Two-way ANOVA was used for (E) and (G), one-way ANOVA was used for (F), with ****p<0.0001, ***p<0.001, ** p<0.01, * p <0.05.

Figure S5

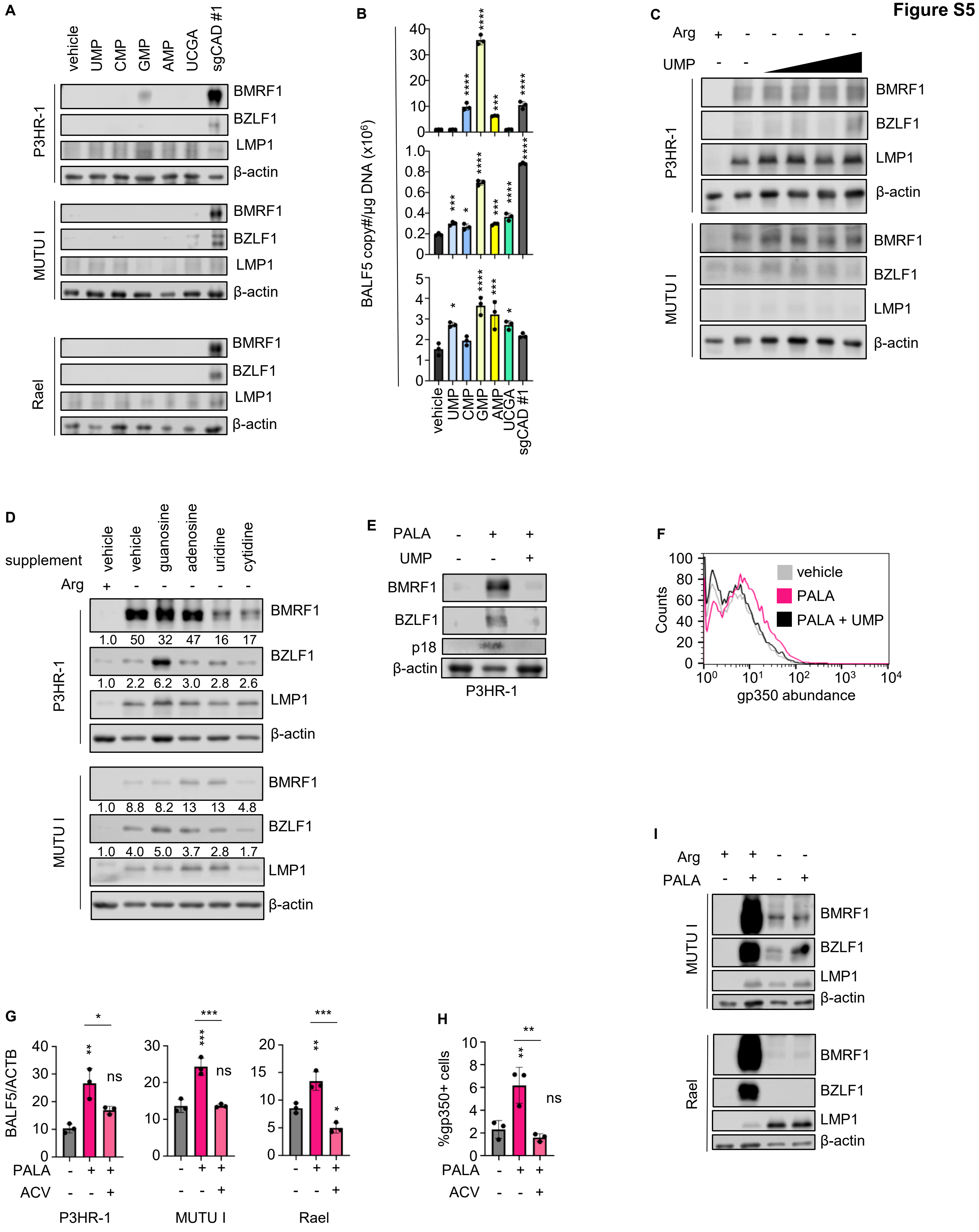

A
vehicle
UMP
CMP
GMP
AMP
UCGA
sgCAD #1
P3HR-1
MUTU I
Rael
BMRF1
BZLF1
LMP1
β-actin
B
BALF5 copy#/μg DNA (x10^6)
C
Arg
UMP
D
supplement
vehicle
guanosine
adenosine
uridine
cytidine
1.0 50 32 47 16 17
1.0 2.2 6.2 3.0 2.8 2.6
1.0 8.8 8.2 13 13 4.8
1.0 4.0 5.0 3.7 2.8 1.7
E
PALA
p18
P3HR-1
F
Counts
gp350 abundance
vehicle
PALA
PALA + UMP
G
BALF5/ACTB
ACV
ns
H
%gp350+ cells
I

Figure S5. ***De novo*** **pyrimidine biosynthesis maintains EBV latency, related to Figure 4**

(A) Immunoblot analysis of WCL from P3HR-1 cells that were cultured in RPMI supplemented with the indicated nucleotide monophosphates to a total concentration of 50µg/ml. Shown at right as a + control or lysates from CAD depleted cells, taken at 48 hours after CAD-targeting sgRNA expression. (B) qPCR analysis of intracellular EBV genome copy number of cells as in (A). Shown are the mean ± SD values from n=3 replicates, normalized by DNA content. (C) Immunoblot analysis of WCL from cells cultured in arginine replete or arginine free media with 0µg/ml, 0.5µg/ml, 5µg/ml, 50µg/ml or 500µg/ml UMP supplementation for 5 days. (D) Immunoblot analysis of WCL from EBV+ P3HR-1 or MUTU I cells cultured in replete or arginine-free media supplemented with 138 µM guanosine, 144 µM adenosine, 154µM uridine, or 155 µM cytidine supplementation for 5 days. Nucleotide concentrations were chosen to be equal molarity with nucleotide monophosphates used in Figure 4E. (E) Immunoblot analysis of WCL from P3HR-1 cells cultured in media with 250µM PALA and with vehicle versus 50µg/ml UMP for 4 days, as indicated. (F) FACS analysis of plasma membrane gp350 abundance in cells as in (E). (G) Analysis of PALA effects on EBV genome copy number. Shown are the mean ± SD *ACTB* normalized intracellular EBV genome copy number of the indicated Burkitt cells cultured in replete media with PALA (250µM) and acyclovir (100 µg/ml) for 4 days, as indicated. (H) Mean percentages ± SD of gp350+ P3HR-1 cells following culture ± 250µM PALA for 4 days, in the absence or presence of acyclovir. (I) Immunoblot analysis of WCL from EBV+ MUTU I or Rael cells cultured in arginine replete or free media, in the absence or presence of 250µM PALA for 4 days. (One-way ANOVA analysis was performed for (B), (G), and (H). *, $p<0.05$, ***$p<0.001$, ****$p<0.0001$. Blots are representative of n=3 replicates.

Figure S6

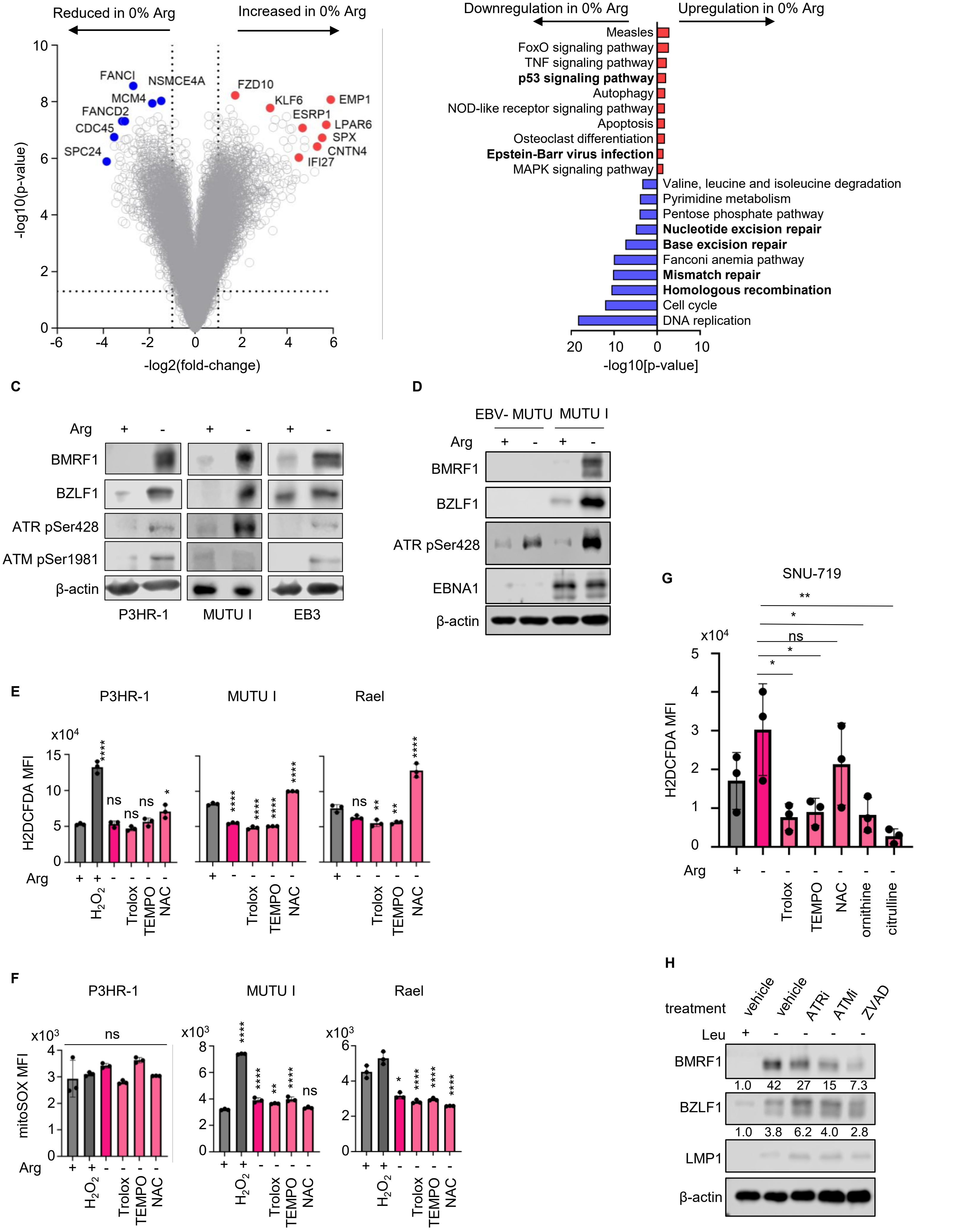
A
Reduced in 0% Arg
Increased in 0% Arg
FANCI
NSMCE4A
MCM4
FANCD2
CDC45
SPC24
FZD10
KLF6
EMP1
ESRP1
LPAR6
SPX
CNTN4
IFI27
-log10(p-value)
-log2(fold-change)
B
Downregulation in 0% Arg
Upregulation in 0% Arg
Measles
FoxO signaling pathway
TNF signaling pathway
p53 signaling pathway
Autophagy
NOD-like receptor signaling pathway
Apoptosis
Osteoclast differentiation
Epstein-Barr virus infection
MAPK signaling pathway
Valine, leucine and isoleucine degradation
Pyrimidine metabolism
Pentose phosphate pathway
Nucleotide excision repair
Base excision repair
Fanconi anemia pathway
Mismatch repair
Homologous recombination
Cell cycle
DNA replication
-log10[p-value]
C
Arg
BMRF1
BZLF1
ATR pSer428
ATM pSer1981
β-actin
P3HR-1
MUTU I
EB3
D
EBV- MUTU
MUTU I
Arg
BMRF1
BZLF1
ATR pSer428
EBNA1
β-actin
E
P3HR-1
MUTU I
Rael
x10^4
H2DCFDA MFI
Arg
H2O2
Trolox
TEMPO
NAC
F
P3HR-1
MUTU I
Rael
x10^3
mitoSOX MFI
Arg
H2O2
Trolox
TEMPO
NAC
G
SNU-719
x10^4
H2DCFDA MFI
Arg
Trolox
TEMPO
NAC
ornithine
citrulline
H
treatment
vehicle
vehicle
ATRi
ATMi
ZVAD
Leu
BMRF1
1.0 42 27 15 7.3
BZLF1
1.0 3.8 6.2 4.0 2.8
LMP1
β-actin

Figure S6. Arginine restriction effects on DNA damage, related to Figure 5.

(A) Arginine restriction effects on EBV+ Burkitt host gene expression. Volcano plot analysis of transcriptomic analysis of P3HR-1 cells cultured in replete versus arginine free media for 5 days from n=3 replicates, as in Figure 2D. (B) KEGG pathway analysis of the top ten pathways enriched within genes differentially expressed in (A). (C) Analysis of arginine restriction effects on ATM and ATR phosphorylation as a readout of their activity. Shown are immunoblots of WCL from EBV+ P3HR-1, MUTU I or EB3 cells cultured in replete or arginine free media for 5 days. (D) Cross-comparison of arginine restriction effects on DNA damage of EBV+ vs EBV- Burkitt cells. Shown are immunoblots of WCL from EBV- versus EBV+ MUTU I cells cultured for 5 days in replete versus arginine free media. (E) Analysis of arginine restriction effects on Burkitt ROS levels. Mean ± SD total ROS levels from n=3 replicates, as judged by H2DCFDA fluorescence signal intensity of the indicated EBV+ Burkitt cells cultured in replete of arginine-free media ± the antioxidants NAC (10mM), Trolox (50μM) or TEMPO (12.5μM) for 5 days. Cells treated with H_2O_2 (50μM) for 1 hr as a positive control. (F) Analysis of arginine restriction effects on Burkitt mitochondrial ROS levels. Shown are mean ± SD of mitochondrial ROS as judged by n=3 replicates of FACS analysis of mitoSOX signal intensity in cells cultured as in (E). (G) Analysis of arginine restriction effects on EBV+ gastric carcinoma ROS levels. Mean ± SD total ROS levels from n=3 replicates, as judged by H2DCFDA fluorescence signal intensity of SNU-719 cells cultured in replete of arginine-free media with NAC, Trolox, TEMPO, or the arginine metabolites ornithine or citrulline (1.15mM) for 5 days. (H) Immunoblot analysis of WCL from P3HR-1 cells grown in replete or leucine-free RPMI and treated with vehicle, VE-822 (ATRi, 5 μM), KU-60019 (ATMi, 5μM) or Z-VAD-FMK (20 μM) for 5 days One-way ANOVA was performed for all statistical analysis. ****p<0.0001, ***p<0.001, **p<0.01, *p<0.05. Blots are representative of n=3 replicates.

Figure S7

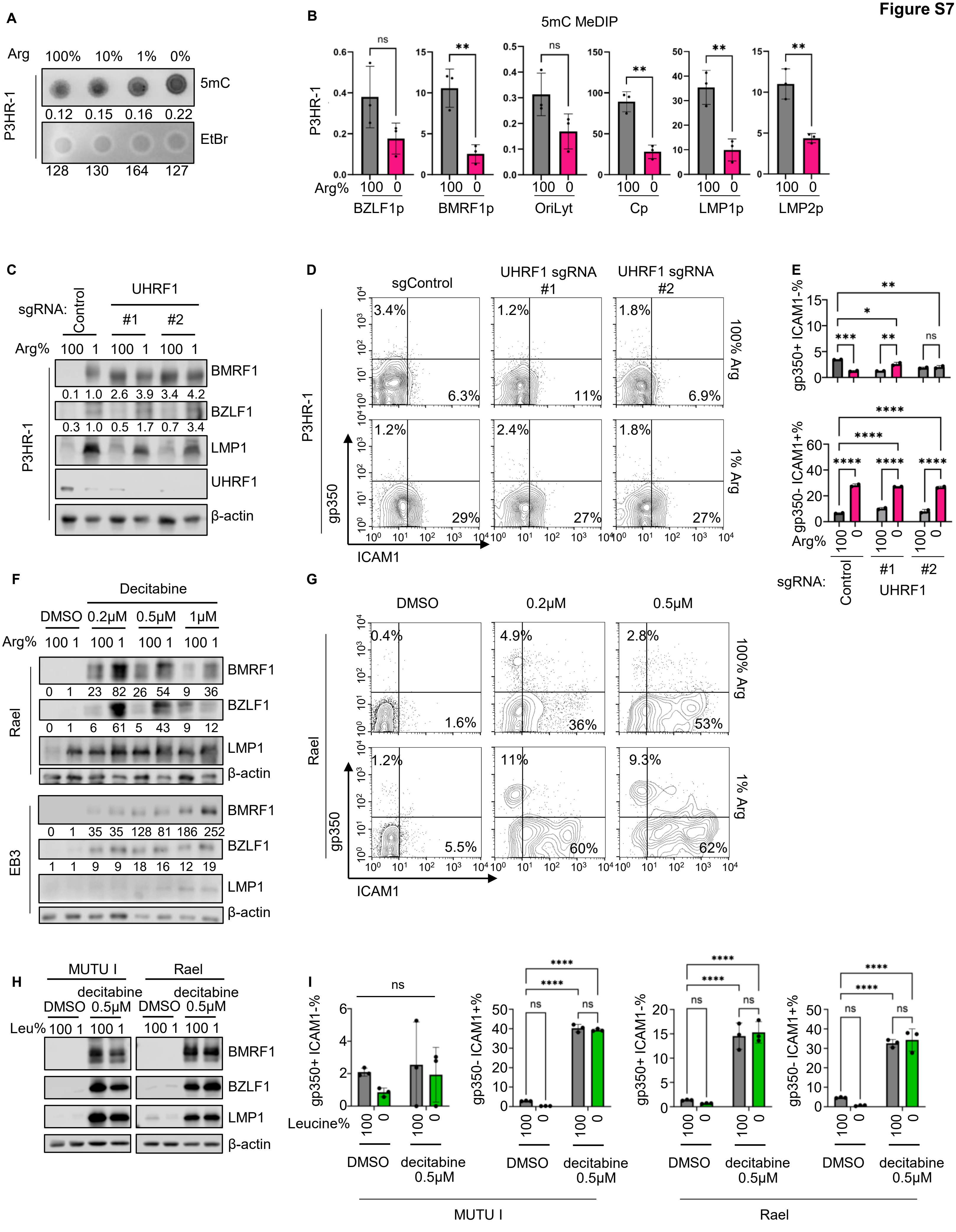
A
Arg 100% 10% 1% 0%
P3HR-1
5mC
0.12 0.15 0.16 0.22
EtBr
128 130 164 127
B
5mC MeDIP
P3HR-1
Arg% 100 0
BZLF1p
BMRF1p
OriLyt
Cp
LMP1p
LMP2p
C
sgRNA: Control UHRF1 #1 #2
Arg% 100 1 100 1 100 1
BMRF1
0.1 1.0 2.6 3.9 3.4 4.2
BZLF1
0.3 1.0 0.5 1.7 0.7 3.4
LMP1
UHRF1
β-actin
D
sgControl
UHRF1 sgRNA #1
UHRF1 sgRNA #2
3.4% 6.3% 1.2% 11% 1.8% 6.9%
1.2% 29% 2.4% 27% 1.8% 27%
100% Arg
1% Arg
gp350
ICAM1
E
gp350+ ICAM1-%
gp350- ICAM1+%
sgRNA: Control #1 #2 UHRF1
F
Decitabine
DMSO 0.2μM 0.5μM 1μM
Arg% 100 1 100 1 100 1 100 1
Rael
BMRF1
0 1 23 82 26 54 9 36
BZLF1
0 1 6 61 5 43 9 12
LMP1
β-actin
EB3
BMRF1
0 1 35 35 128 81 186 252
BZLF1
1 1 9 9 18 16 12 19
LMP1
β-actin
G
DMSO 0.2μM 0.5μM
Rael
0.4% 1.6% 4.9% 36% 2.8% 53%
1.2% 5.5% 11% 60% 9.3% 62%
100% Arg
1% Arg
gp350
ICAM1
H
MUTU I Rael
DMSO decitabine 0.5μM
Leu% 100 1 100 1 100 1 100 1
BMRF1
BZLF1
LMP1
β-actin
I
gp350+ ICAM1-%
gp350- ICAM1+%
Leucine%
DMSO
decitabine 0.5μM
MUTU I
Rael

Figure S7. Arginine restriction and DNA hypomethylation combinatorial effects on EBV reactivation, related to Figure 5.

(A) 5mC dot blot analysis of DNA extracted from P3HR-1 cells that were cultured in RPMI with the indicated arginine levels for 5 days. Membranes were stained with ethidium bromide as load controls. Each dot contains 500 ng DNA. (B) 5mC MeDIP analysis of chromatin from P3HR-1 cells cultured in arginine free versus replete RPMI media for 5 days. 100 μg/ml acyclovir was added to prevent lytic DNA replication. Mean ± SD values from n = 3 replicates are shown. (C) Immunoblot analysis of WCL from P3HR-1 cells that expressed control or *UHRF1*-targeting sgRNA and were cultured in arginine restricted (1% Arg) vs replete media. Cells were induced to express sgRNA for 3 days and then cultured in the indicated media for 5 days. (D) FACS analysis of plasma membrane gp350 and ICAM-1 expression on cells treated as in (C). (E) Mean ± SD percentages of gp350+ and ICAM-1+ cells from n=3 replicates as in (D).. (F) Immunoblot analysis of WCL from EBV+ Rael or EB3 cells that were cultured in media containing indicated DCB concentrations and then cultured in 1% Arg or 100% Arg media for 5 days. (G) FACS analysis of plasma membrane gp350 and ICAM-1 levels in Rael cells treated as in (F). (H) Analysis of leucine restriction and decitabine effects on Burkitt EBV reactivation. Immunoblot analysis of WCL from EBV+ MUTU I or Rael cells that were cultured in media containing DMSO vehicle vs decitabine (0.5μM) and in media with the indicated leucine concentrations for 5 days. (I) Mean ± SD percentages of gp350+ and ICAM-1+ cells from n=3 replicates of cells cultured as in (H). Student's T test was performed for (B) and two-way ANOVA was performed for (E) and (I). ****p<0.0001, ***p<0.001, **p<0.01, *p< 0.05.
