# Supplementary material for: Arginine metabolism supports de novo pyrimidine biosynthesis to block DNA damage and maintain Epstein-Barr virus latency
Source: mBio. 2026 Jun 15;17(7):e00933-26. doi: 10.1128/mbio.00933-26 (PMC13343950; doi:10.1128/mbio.00933-26)
Supplement: Table S4 — Amino acid stock solutions used in amino acid supplementation. [file mbio.00933-26-s0005.docx]

| **Amino acid** | **Concentration (mg/L) in RPMI** | **mM in RPMI** | **Stock Solution** | **Solvent** | **Dilution factor** |
| --- | --- | --- | --- | --- | --- |
| L-Histidine | 15 | 0.096774 | 15mg/ml | H_2_O | 1000x |
| L-Isoleucine | 50 | 0.381679 | 25mg/ml | H_2_O | 500x |
| L-Leucine | 50 | 0.381679 | 5mg/mL | H_2_O | 100x |
| L-Lysine hydrochloride | 40 | 0.218579 | 40mg/mL | H_2_O | 1000x |
| L-Methionine | 15 | 0.100671 | 15mg/mL | H_2_O | 1000x |
| L-Phenylalanine | 15 | 0.090909 | 15mg/ml | H_2_O | 1000x |
| L-Threonine | 20 | 0.168067 | 20mg/ml | H_2_O | 1000x |
| L-Tryptophan | 5 | 0.02451 | 5mg/ml | H_2_O | 1000x |
| L-Valine | 20 | 0.17094 | 2mg/ml | H_2_O | 100x |
| L-Arginine | 200 | 1.149425 | 100mg/ml | H_2_O | 500x |
| L-Asparagine | 50 | 0.378788 | 25mg/ml | H_2_O | 500x |
| L-Aspartic acid | 20 | 0.150376 | 6.65mg/ml | H_2_O | 333x |
| L-Cystine 2HCl | 65 | 0.207668 | 50mg/ml | 1N HCl | 770x |
| L-Glutamic Acid | 20 | 0.136054 | 20mg/ml | H_2_O | 1000x |
| L-Glutamine | 300 | 2.054795 | 200mM | commercial | 100x |
| Glycine | 10 | 0.133333 | 10mg/ml | H_2_O | 1000x |
| L-Hydroxyproline | 20 | 0.152672 | 20mg/ml | H_2_O | 1000x |
| L-Proline | 20 | 0.173913 | 20mg/ml | H_2_O | 1000x |
| L-Serine | 30 | 0.285714 | 30mg/ml | H_2_O | 1000x |
| L-Tyrosine disodium salt dihydrate | 29 | 0.111111 | 29mg/ml | H_2_O | 1000x |

Tabel S4. Amino acid stock solutions used in amin acid supplementation.
